# Sequencing and *de novo* assembly of a near complete *indica* rice genome

**DOI:** 10.1038/ncomms15324

**Published:** 2017-05-04

**Authors:** Huilong Du, Ying Yu, Yanfei Ma, Qiang Gao, Yinghao Cao, Zhuo Chen, Bin Ma, Ming Qi, Yan Li, Xianfeng Zhao, Jing Wang, Kunfan Liu, Peng Qin, Xin Yang, Lihuang Zhu, Shigui Li, Chengzhi Liang

**Affiliations:** 1 State Key Laboratory of Plant Genomics, Institute of Genetics and Developmental Biology, Chinese Academy of Sciences, 1 Beichen West Road No. 2, Beijing 100101, China; 2 College of Life Sciences, University of Chinese Academy of Sciences, Beijing 100049, China; 3 Rice Research Institute, Sichuan Agricultural University, Chengdu 611130, China

## Abstract

A high-quality reference genome is critical for understanding genome structure, genetic variation and evolution of an organism. Here we report the *de novo* assembly of an *indica* rice genome Shuhui498 (R498) through the integration of single-molecule sequencing and mapping data, genetic map and fosmid sequence tags. The 390.3 Mb assembly is estimated to cover more than 99% of the R498 genome and is more continuous than the current reference genomes of *japonica* rice Nipponbare (MSU7) and *Arabidopsis thaliana* (TAIR10). We annotate high-quality protein-coding genes in R498 and identify genetic variations between R498 and Nipponbare and presence/absence variations by comparing them to 17 draft genomes in cultivated rice and its closest wild relatives. Our results demonstrate how to *de novo* assemble a highly contiguous and near-complete plant genome through an integrative strategy. The R498 genome will serve as a reference for the discovery of genes and structural variations in rice.

## Abstract (web summary)

High-quality reference genomes facilitate analysis of genome structure and variation. Here Du *et al*. create a near-complete assembly of the *indica* rice genome by combining single molecule sequencing with mapping data and fosmid sequences and identify genetic variants by comparison with other rice genomes.

Assembly of a high-quality reference genome is the basis for identifying genes and genetic variations, especially structural variations (SVs) which include duplications, insertions, deletions, inversions and translocations. SVs account for more heritable nucleotide variants in a population than single-nucleotide variations and have an important influence on human disease and phenotypic diversity in both animals^1^,^2^ and plants^3^,^4^. Next-generation sequencing technologies have been used in the *de novo* assembly of many plant and animal genomes over the past decade. However, the genomes assembled from short reads are generally draft genomes consisting of up to tens of thousands of contigs, especially in plants, due to high repeat content or low sequencing coverage at complex GC- or AT-rich regions^5^,^6^. Draft genome assemblies often contain fragmented genes, collapsed or redundant repeats and chimeric contigs that confound gene functional assignment and SV detection. Therefore, improving and validating genome assemblies through the application of up-to-date computational and experimental methods is an important foundation-building task for genomics-driven studies. Single molecule, real-time (SMRT) sequencing on the Pacific Biosciences (PacBio) platform generates long reads of up to 40–60 kb with randomly distributed errors^7^. This makes SMRT sequencing very suitable for *de novo* genome assembly under high sequencing depth, and it has been used to assemble complete microbial genomes, high-quality plant draft genomes and significantly improved human and animal reference genomes^8^,^9^,^10^,^11^.

However, although the assembled contig length has been significantly improved with the SMRT sequencing data, the problem of fragmentation in the genome assemblies mentioned above still exists. To create chromosome-scale pseudomolecules in genome assembly, in plants, it is common to use a genetic map to order and orient the assembled contigs/scaffolds, but it does not fill gaps and often leaves many misordered, unoriented and unanchored contigs/scaffolds, as well as potentially unfixed assembled errors. A correctly constructed chromosome sequence is critical for gene or quantitative trait locus mapping and genome-wide association studies through linkage disequilibrium-based analysis. Large-insert DNA fragment libraries have played a critical role in sequencing and assembling all high-quality reference genomes in the past, due to their ability to resolve segmentally duplicated regions^12^. There exist several types of large-insert libraries, of which the fosmid library has many advantages over others, such as its shorter construction time, lower cost and better genome coverage resulting from lower cloning bias. However, the high cost involved in sequencing those libraries has largely limited their usage to a small number of model organisms. Furthermore, a recently developed technology, the Irys single-molecule next-generation mapping system from BioNano Genomics, provides physical maps (genome maps) of hundreds of kilobases (kb) to megabases (Mb) in length based on the distribution of nicking sites of endonucleases. Although the genome maps can be used to scaffold assembled sequences or identify SVs for verifying the assembly quality^13^,^14^, short contigs of up to 100–200 kb are often not matchable to genome maps due to the shortage of nicking sites, which leads to many large gaps in the scaffolds to be resolved by other methods.

Rice (*Oryza sativa*, 2*n*=2*x*=24) is one of the most important crops in the world, providing 19% of daily caloric intake per capita globally^15^. Traditionally, rice is grouped into two subspecies: *indica* and *japonica*, which diverged from their common ancestors ∼0.44 million years ago^16^. The two rice subspecies differ significantly both in morphological traits and at the sequence level, and often show various degrees of intercross hybrid sterility^17^,^18^. The current rice reference genome was sequenced from the *japonica* cultivar Nipponbare (Nip) using the clone-based assembly approach^19^. However, *indica* rice is planted in more areas worldwide than *japonica* rice, has more genetic diversity and can be further subdivided into *indica* and *aus* subpopulations^20^. Although several draft genomes of *indica* rice have been assembled over the past decade^21^,^22^,^23^,^24^, their fragmented or incomplete nature has limited their usage in gene or genetic variation discovery. Therefore, a high-quality reference genome for *indica* cultivars is still required for expanding our knowledge on rice genome function and evolution^25^.

Here, we present a cost-effective method for *de novo* assembly of high-quality reference genomes by leveraging whole-genome shotgun (WGS) SMRT sequencing, pooled fosmid clone sequencing and genetic map construction, and using BioNano genome maps to verify and help correct the assembled sequences. We assemble a highly contiguous and near-complete *indica* rice genome of the cultivar Shuhui498 (R498), a restorer line in a three-line hybrid system. We provide high-quality genome annotations for R498 and identify genetic variations by comparing it to the Nip reference genome. We also use both R498 and Nip as a reference to identify presence/absence variations (PAVs) in 17 other deeply sequenced rice genomes. Our method can effectively assemble high-quality complex genomes. This R498 assembly provides an extra resource for gene discovery and for studying genetic variations in rice.

## Results

### Genome sequencing and construction of super-contigs

We sequenced R498 genomic DNA to generate 47 Gb (∼118 × ) of WGS SMRT subread sequences from 23 cells using P5 polymerase binding and C3 chemistry and 38 cells using P6 polymerase binding and C4 chemistry, with N50 of 11 kb (Supplementary Table 1). The longest 10 Gb sequences (∼25 × genome coverage) had a length of at least 16 kb. We sequenced an F_3_ population of 364 individuals produced from a cross between R498 and Nip via genotyping-by-sequencing (GBS) technology^26^. In all, we generated 26.9 Gb of clean GBS tags of length 2 × 125 bp with an average of 73 Mb sequences for each F_3_ individual (4 × sequencing depth for each tag). We sequenced 564 fosmid pools, each containing ∼1,000 independent clones with insert size of ∼40 kb, to produce 6.3 Gb of GBS tags of length 2 × 125 bp (3 × for each tag on average).

We first used the PBcR^27^ pipeline to correct the raw SMRT sequences, which resulted in 16.2 Gb of self-corrected sequences. We assembled the corrected sequences on two sets of parameters: default low stringency (LS) and high stringency (HS), which resulted in 436 and 471 Mb of sequences, with contig N50 size of 1.1 Mb and 443 kb, respectively (Supplementary Table 2). We also corrected and assembled the raw SMRT sequences with Falcon and CANU pipelines, resulting in an assembly size of 397 and 405 Mb, and contig N50 size of 516 and 900 kb, respectively (Supplementary Table 2). The PBcR LS assembly was used as reference for the construction of genetic map due to its largest N50. The constructed genetic map consisted of 12 linkage groups (LGs), which anchored and ordered 497 contigs, with a total sequence length of 355.9 Mb. The fosmid GBS tags were aligned to the PBcR corrected SMRT sequences to retrieve 800 Mb (∼20 × of the pooled fosmid insert size) of the best aligned sequences into each fosmid pool. Each pool of the selected sequences was assembled with Falcon separately and the contigs shorter than 10 kb were discarded. The resulting fosmid contigs were pooled together to a total size of 39.7 Gb, with N50 size of 42 kb, and max length of 616.8 kb (Supplementary Table 2). It is worth mentioning that all the fosmid contigs were generated from the WGS SMRT data, but not from fosmid clone sequences, which minimized sequence variations introduced in the cloning process. We aligned all the fosmid contigs to both PBcR assemblies and found that <5% of the aligned fosmid contigs were chimeric (Supplementary Fig. 1a) and the average alignment identity was >99%, suggesting that the fosmid contigs were of high quality for connecting the WGS contigs.

**Figure 1:**
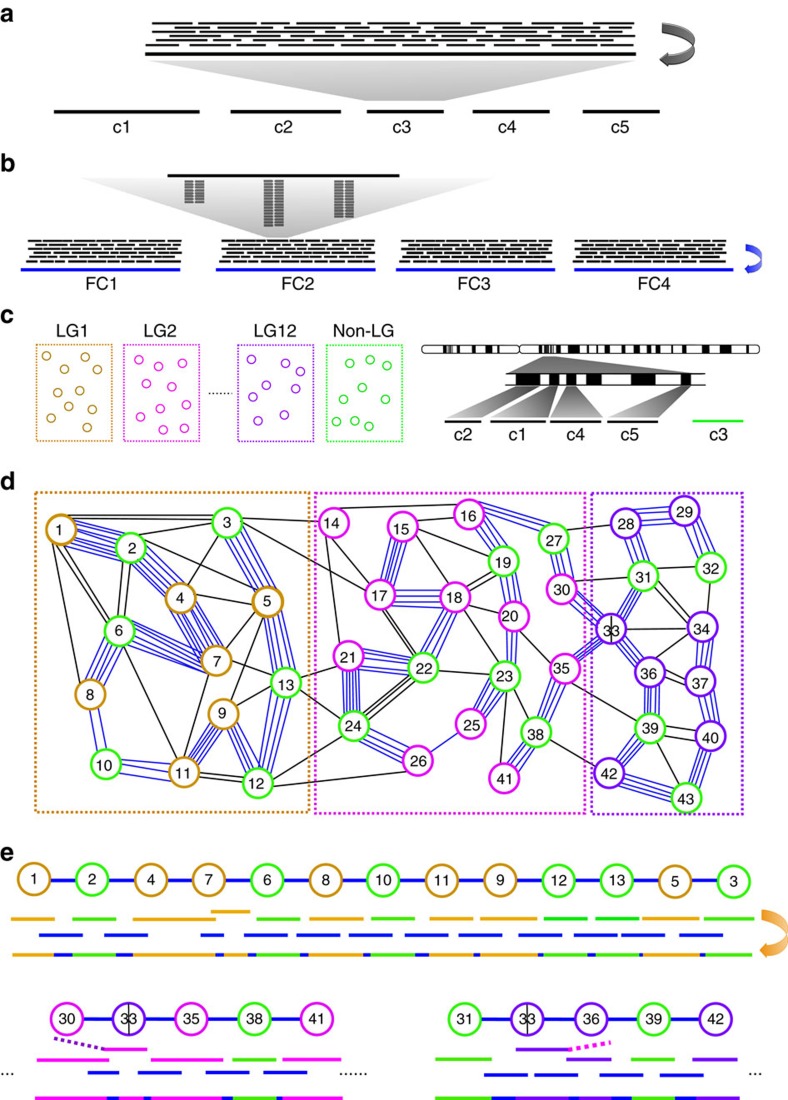
Schematic depiction of the construction of super-contigs. (**a**) Whole-genome assembly from corrected SMRT sequences (thin black lines) generates a set of WGS contigs (c1–c5). (**b**) Sequence tags (stacked short lines) from each fosmid pool are used to retrieve corrected PacBio sequences (black lines), which are then assembled into fosmid contigs (for example, FC1–FC5). (**c**) Many of the WGS contigs are grouped together and anchored onto chromosomes based on their linkage relationship (LG1–LG12). For example, c1, c2, c4 and c5 are anchored onto a chromosome, but c3 is not (in the non-LG group). Note that c2 and c4 are close to c1 physically, but c5 is not close to them. (**d**) A simplified overlap graph is constructed using anchored WGS contigs in LG1, LG2 and LG12 and unanchored WGS contigs in the non-LG group as nodes and fosmid contigs as edges (blue lines). A sequence overlap between a WGS contig and a fosmid contig must be at least 5 kb to form a connection. Two WGS contigs can be connected by multiple fosmid contigs. (**e**) Three most reliable paths from (**d**), including two partial ones, are selected as described in the Methods section to build super-contigs. The blue lines represent fosmid contigs. The other coloured lines represent WGS contigs. The WGS contigs (including unanchored ones in green) on each path are connected to the best aligned fosmid contigs to form super-contigs. The overlap between two WGS contigs (for example, nodes 4 and 7) causes negative edge length as described in the Methods section. In such cases, the fosmid sequences were used for the overlapping regions to form the super-contig. In all other cases, the WGS contig sequences were used for the overlapping regions. Node 33 represents a chimeric WGS contig, which is split into two parts to be used separately in two super-contigs. Dashed lines represent alignment overhangs.

As fully described in the Methods section, the WGS contigs on the genetic map were connected or extended into super-contigs iteratively by selecting the best paths on the overlap graphs that were constructed using WGS contigs as nodes and fosmid contigs as edges (Fig. 1). We minimized connection errors by using each WGS contig only once and carefully prioritize the node pairs to be connected at any stage (Supplementary Note 1). Whole-genome assembly often produces misassembled chimeric contigs. Those contigs were captured during the merging process if they were aligned with an overhang of >1 kb to its adjacent contigs with high sequence identity (>98%) and the overlap was also supported by fosmid contigs. The identified chimeric contigs were split into multiple contigs at the detected joining positions, and the cleaved sequences longer than 30 kb were reused elsewhere as new WGS contigs (Fig. 1e).

For building super-contigs, we found that although the LS contigs had higher N50 size, the HS contigs were better due to their higher accuracy (Supplementary Note 1). The resulting genome based on the HS contigs consisted of 17 super-contigs (a total of 390.6 Mb), with seven chromosomes assembled into single super-contigs and the remaining five each assembled into two super-contigs. We detected a long stretch of the telomeric sequence 5′-TTTAGGG-3′ at all chromosome ends, suggesting the completion of every chromosome arm at the end.

The 17 super-contigs incorporated 1,693 PBcR HS contigs and 1,676 fosmid connections, including 378 connections supported by both sequence overlaps between WGS contigs and fosmid contigs (Fig. 1e). During the connection process, 36 and 13 chimeric contigs were identified and split in the PBcR LS and HS assemblies, respectively. Of the 497 anchored LS contigs on the genetic map, we found that 127 contigs (a total of 45.3 Mb) were repositioned relative to other contigs and 272 contigs (a total of 73.7 Mb) were reoriented on the super-contigs.

### Correction of errors in the super-contigs

We obtained a total length of 99 Gb (250 × ) of BioNano single-molecule maps (>100 kb) with N50 size of 202 kb, which were *de novo* assembled to 453 genome maps with a total size of 406 Mb and N50 of 1.22 Mb (Supplementary Data 1). The relative short length of the genome maps was caused by the existence of common breaking points in the raw single-molecule maps^14^. We also conducted a hybrid assembly by combining the PBcR LS contigs and the BioNano maps, which generated sequence scaffolds that were represented by 225 genome maps with a total size of 408 Mb and N50 size of 2.48 Mb. Since short contigs (with <8 nicking sites) were not aligned to genome maps, the scaffolds contained many gaps of up to a few hundred kb. Notably, it is possible to build super-contigs under the guidance of hybrid genome maps using the method described above, but the genetic map is still required to anchor the super-contigs onto chromosomes. Therefore, in this study we only used the genome maps to assess the quality of the super-contigs constructed under the guidance of genetic map and to help correct potential assembly errors in the super-contigs.

For convenience we added 10 kb of ‘N's between each pair of contigs on the same chromosome to connect them into pseudomolecules and aligned the *de novo* assembled genome maps to them. The genome maps (446 of 453) covered 96.62% of the 12 pseudomolecules with 226 uncovered gaps >0 bp (a total of 13.2 Mb with N50 size of 125 kb and max 495 kb; Supplementary Fig. 2). For quality check, we first examined the most error-prone regions in the genome: centromeres and subtelomeres. The successfully assembled seven centromeres in R498 were confirmed to be correct but none of the five gaps in the centromeric regions were covered completely by a single map (Supplementary Fig. 3). Of the 24 assembled chromosome ends, 21 subtelomeres were also confirmed correct (Supplementary Fig. 4). There were potentially missing subtelomeric repeats in the distal end of chromosome 3, 10 and 11. The nucleolus organizer regions were found to be located at the start of two pseudomolecules 9 and 10, which contained six and nine assembled copies of 17S-5.8S-25S ribosomal RNA genes (rDNAs), respectively (Supplementary Fig. 4). One genome map was aligned to chromosome 9, which contained 22 copies of rDNAs (Supplementary Fig. 4). We found 25 maps aligned to the start of chromosome 10, of which 24 (a total of 17.1 Mb) appeared to be rDNA-only maps (Supplementary Fig. 4), and the only map that was extended to non-rDNA region contained a tandem array of 40 rDNAs. We identified telomere repeats immediately adjacent to rDNAs in many SMRT reads, confirming the chromosomal end location of rDNAs. Due to their sequence complexity, we did not try to fix the missing sequences in these two rDNA regions.

Next, we identified SVs in the pseudomolecules as potential assembly or connection errors compared with the genome maps (Supplementary Fig. 5 and Supplementary Table 13). Excluding the two rDNA regions, we found 44 insertions and deletions (indels) longer than 10 kb (max length 175 kb), including 17 insertions (a total of 350 kb) and 17 deletions (a total of 1.6 Mb), and 63 indels of 1–10 kb (a total of 410 kb) in the pseudomolecules. The genome maps fully covered 1,621 (96.72%) connecting regions, of which 74 were present in 54 indels (34 indels >10 kb), indicating a max error rate of 4.57% for misconnections (note that not all connections were wrong in the indels involving multiple connections). As a comparison, the other 53 indels (10 of them >10 kb) were present in the original PBcR HS contigs. We found that all the potential misconnections were local errors mostly with missing repetitive sequences, that is, none of the genome maps was aligned to two non-adjacent genome locations.

Of the indels >10 kb, 30 were corrected by choosing an alternative route of the correct length on the overlapping graph with matching nicking sites to replace the erroneously incorporated contigs (Supplementary Fig. 5). The 14 uncorrected indels (a total size of 1 Mb), including 11 in connecting regions, were either long tandem repeats such as one with >60 kb repeat unit on chromosome 5 or complex repeats such as those in the subtelomere regions (Supplementary Fig. 6). To fix the potential indel errors of 1–10 kb, we aligned the corrected SMRT reads to each region and conducted local assembly with the aligned sequences. We found that 46 of the regions were reassembled correctly by comparing the newly assembled contigs to the genome maps, so we used the new contigs to replace the incorrect sequences. The remaining 17 indels, including 3 in connecting regions, were not changed because the local reassembly still generated the same sequences. After correction of the indels, the total number of connecting regions was reduced to 1,652, which connected 1,669 HS contigs in the final pseudomolecules.

Finally, the assembled genome sequences were corrected by Quiver^8^, which fixed minor sequence errors up to 1–2 kb. We then aligned 38.7 Gb (∼98 × ) of Illumina short reads to the pseudomolecules (genome coverage of 99.88%) to identify and fix 40,784 homozygous indels of 1–2 bp, which were resulted from the non-random indel errors in homopolymer runs in SMRT sequences. The resulting genome sequences were 390,322,188 bp (including 50 kb of ‘N's; GC content, 43.57%). We realigned the genome maps to the pseudomolecules (Supplementary Data 2) to recompute the coordinates of the error-prone regions (Supplementary Table 3).

### Assessment of the sequence quality

We estimated the base error rate in the genome to be <0.0017% with both Illumina DNA short reads and RNA sequencing (RNA-seq) data (Supplementary Table 4). This error rate was well below one error per 10,000 bases, a quality standard used for human genome^28^ and other high-quality genomes such as Nipponbare^19^. The heterozygous single-nucleotide polymorphisms (SNPs) and indels represented genome heterozygosity of 0.0038% in our sequenced R498 samples, suggesting that the R498 genome was highly homozygous.

The fosmid contigs contributed a total of 11.9 Mb (3.05%) sequences in the final pseudomolecules, with N50 size of 13.9 kb and a max of 81.9 kb for a single connecting region. Using Illumina short reads, the error rate in the connecting regions (with 98.21% being covered) were estimated to be 0.0071%, which was slightly larger than the whole-genome average, most likely due to the repetitive nature of these regions. Using the pseudomolecules as reference, we found that the average base accuracy of all the fosmid contigs was 99.33% and comparable to that of the WGS contigs (Supplementary Fig. 1b). We also found that the chimeric fosmid contigs were randomly distributed throughout the genome (Supplementary Fig. 1c). These results further confirmed that the potential errors introduced by the fosmid contigs were minimal.

The PBcR HS assembly was found to be highly redundant compared with the Falcon or CANU assemblies, but had the highest sensitivity (99.75%) and the lowest error rate (0.57%) (Supplementary Note 2 and Supplementary Table 5). Of the 471.1 Mb sequences in the PBcR HS assembly, only 3 Mb of centromeric sequences and 1 Mb of unclassified sequences were not mapped to the pseudomolecules (Supplementary Table 2). Almost all the unclassified contigs were found to be assembled from partially corrected sequences during PBcR self-correction, as suggested by their low coverage of Illumina short reads (Supplementary Fig. 7). Based on these results and the comparison results to genome maps, we estimated that <1% of the R498 genome sequences were missing in the pseudomolecules (Supplementary Note 3).

### Assembly of two organelle DNAs

The R498 chloroplast DNA (cpDNA) and mitochondrial DNA (mtDNA) were initially connected into two misassembled contigs of 593 and 618 kb, respectively. We selected the corrected PacBio reads that were aligned to these two contigs with a minimum aligned length of 3 kb and a minimum alignment identity of 98% for reassembly. The cpDNA was directly assembled into a circular contig containing a non-redundant sequence of 134.5 kb. The mtDNA was assembled into a circular DNA after several rounds of extension and local reassembly, and connection with fosmid contigs, as described in the Methods section. The resulting non-redundant mtDNA was 527.1 kb, which is 36.5 kb longer than that of Nip (Table 1). We found that the Nip mtDNA was potentially misassembled, most likely due to the complex internal repeat structures that included three sets of large repeats longer than 20 kb (Supplementary Fig. 8). Comparison with genome maps showed that the cpDNA was fully covered by genome maps, but unfortunately the mtDNA was only partially covered by one map of 117 kb.

### Gene and repeat annotation

We predicted 38,714 high-confidence protein-coding genes in R498 through an evidence-based gene prediction pipeline^29^ by combining known proteins, experimentally cloned cDNAs/expressed-sequence tags (ESTs) and assembled transcripts from rice RNA-seq data (see Methods for detailed description). Using RNA-seq data from five tissues types of R498 (Supplementary Table 1), we found that 32,393 (83.67%) of these genes were expressed in at least one of the samples. For unbiased gene comparison between R498 and Nip, we also used the same method to annotate 36,775 protein-coding genes in Nip (Supplementary Table 6). We assigned Pfam motifs or functions to 84.82% and 88.06% of R498 and Nip genes, respectively (Supplementary Table 7). The mutual comparison among our predicted Nip gene set, MSU7 set and RAP set (http://rapdb.dna.affrc.go.jp/) showed that these three gene sets are largely similar to each other with extra unshared genes in each set (Supplementary Fig. 9). Since all our predicted genes have protein homology, cDNA/EST or RNA-seq data as evidence, and each exon were predicted based on sequence alignment, these extra genes in our annotation are very likely true genes that are missing in the other gene sets.

We annotated the repetitive sequences in both R498 and Nip by combining *de novo* prediction and homology-based search at both the DNA and protein levels. The repetitive sequences account for 42.05% and 40.43% of the R498 and Nip genomes, respectively (Supplementary Table 8). Our annotation of 98.9 Mb long terminal repeat (LTR)-retrotransposons in Nip corresponds well to the previous estimation of at least 97 Mb in the Nip genome^30^. We annotated more repeated sequences in Nip than MSU7 annotation (34.23%), probably owing to the use of multiple repeat libraries in our method.

### Whole-genome comparison to Nip reference genome

**Figure 2:**
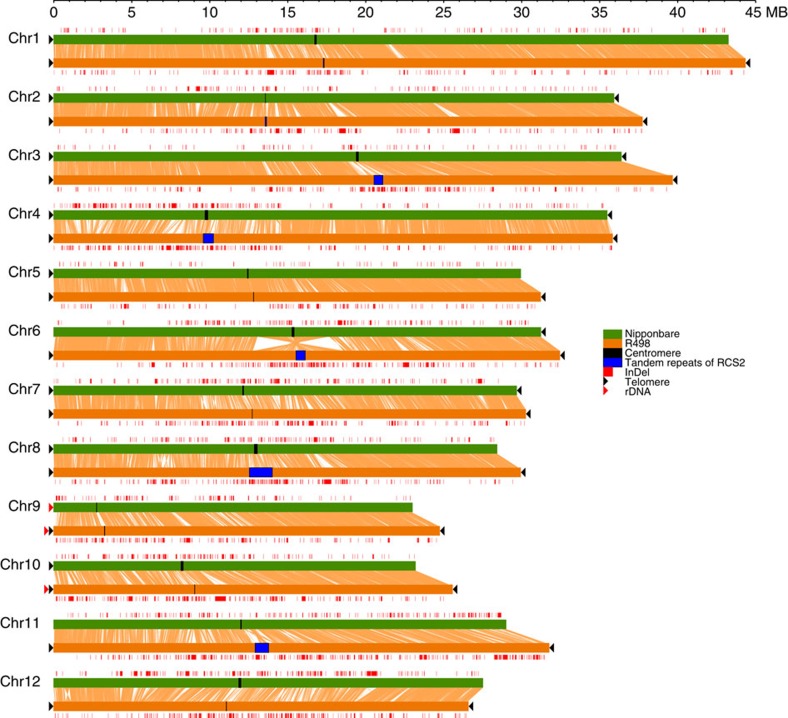
Whole-genome comparison of R498 and Nip. The aligned regions are represented by the crossing lines between each pair of pseudomolecules. A large inversion is observable on chromosome 6. Red rectangles above or below the pseudomolecules represent PVs (≥500 bp) relative to each other. Black rectangles indicate the position of centromere-surrounding sequences defined in Nip reference genome (http://rice.plantbiology.msu.edu/annotation_pseudo_centromeres.shtml), and blue rectangles represent the regions containing centromere-specific tandem repeats of RCS2 in R498. Left or right black arrows at the end of each pseudomolecule indicate the presence of telomere repeats. Red arrows indicate the locations of rDNAs at the start of chromosomes 9 and 10.

The R498 assembly was 17 Mb longer and contained fewer gaps than the current Nip reference genome MSU7 (http://rice.plantbiology.msu.edu, release 7, 373 Mb) (Table 1). Except chromosome 12, all other 11 chromosomes of R498 are longer than Nip. Comparing to Nip, R498 assembly has more completed centromere regions (7 versus 2) (Supplementary Table 9) with considerable divergence between them (Supplementary Fig. 10), more telomeres (24 versus 13; Table 1), more assembled rDNAs at the beginning of chromosomes 9 and 10 (Supplementary Table 10) and fewer cpDNAs and mtDNAs (Supplementary Data 3). We aligned the R498 genome sequences to Nip and found their high chromosome-level similarity, with 313.5 Mb (80.31%) of R498 sequences aligned to 307.8 Mb (82.73%) of Nip sequences in syntenic blocks, and also significant sequence divergence with more than two and half million SNPs (Fig. 2 and Table 2). We identified a large number of SVs between the two genomes. The most remarkable difference between the two genomes was an inversion across the centromere from 12.7 to 18.5 Mb on chromosome 6 (Fig. 2, Supplementary Table 11 and Supplementary Fig. 11a). Both boundaries of this inversion in R498 were confirmed by genome maps (Supplementary Fig. 11b). The alignments of Nip PacBio sequences (http://schatzlab.cshl.edu/data/ectools/) to Nip genome suggested that the Nip sequences were probably also correct. We also found three smaller inversions of a few hundred kilobase whose boundaries were all validated with genome maps in R498 (Supplementary Fig. 11c,d). We found 8,402 presence variations (PVs) in R498 (a total of 66 Mb, max 484.9 kb) and 7,119 PVs in Nip (a total of 39.3 Mb, max 228.5 kb) at least 500 bp, which include 170 and 92 PVs that are at least 50 kb in R498 and Nip, respectively (Supplementary Data 4). As expected, we identified much more large PAVs than those reported previously^23^,^24^ owing to the completeness of the genome. A large PV in Nip was found on chromosome 12 from 20,108,154 to 20,783,761 bp. Further inspection revealed a potential misassembly in the Nip genome from 20,460 to 20,705 kb, which contained a piece of 245 kb mitochondria-like DNA, which was aligned to Nip mtDNA with >99.1% identity in interleaved blocks (Supplementary Data 3).

**Figure 3:**
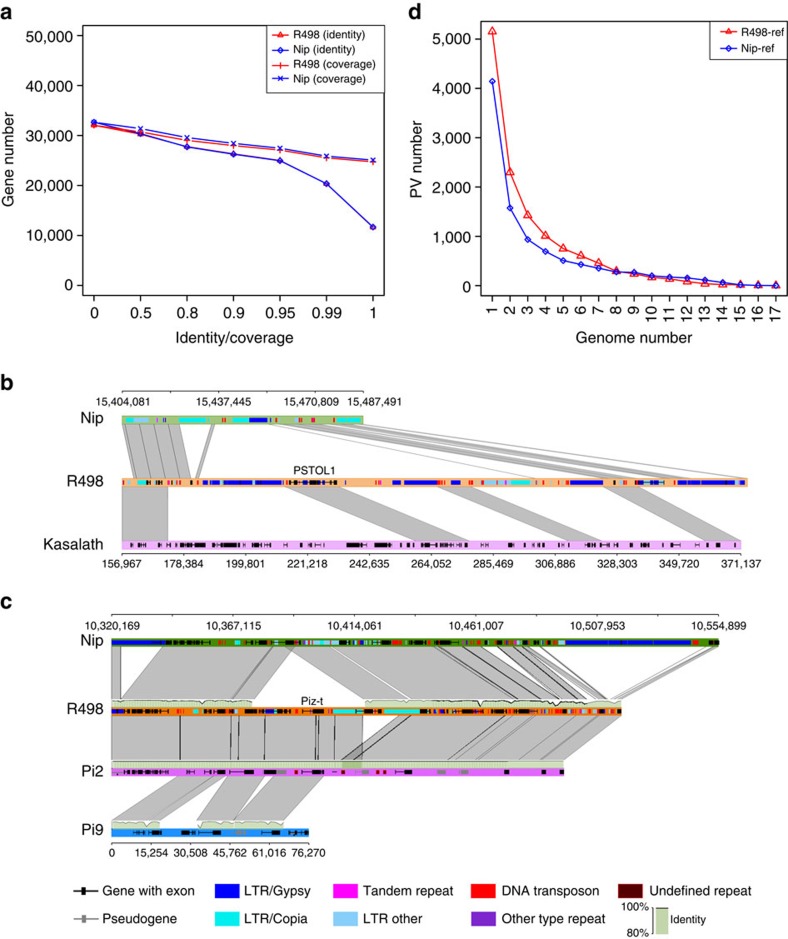
Comparison of genes and PAVs among rice genomes. (**a**) Line plot showing the number of homologous genes between R498 and Nip. The proteins coded by genes in R498 and Nip were BLASTed against each other, and the mutual sequence coverage and alignment identity of the best-matched protein pairs was computed. The total number of genes compared was: R498, 38,714; Nip 36,775. The *x* axis indicates the percentage of either coverage or identity as the threshold. The *y* axis indicates the number of the genes in R498 or Nip that are aligned to best-matched homologous genes in the other genome with coverage or identity above the threshold. (**b**) Synteny view of the *PSTOL1* gene region between R498 (Chr12: 14,818,743–15,035,152 bp), Nip and Kasalath (GenBank accession no. AB458444.1). Different amplification or loss of LTR retrotransposons between R498 and Nip is evident. (**c**) Synteny view of rice blast resistance gene locus for *Piz-t* in R498 (Chr6: 10,079,870–10,277,132 bp), *Pi2* from *indica* cultivar C101A51 and *Pi9* from *O. minuta* (GenBank accession nos DQ352453 and DQ285630) to Nip on chromosome 6. This region is composed of multiple NBS-LRR domain genes, transposons and pseudogenes. Several PAVs of 13–65 kb were shown, which contain different type of LTR transposons. (**d**) Statistics of the PVs on R498 and Nip that are shared by one or more of the 17 draft genomes. The names and data sources of the 17 draft genomes were listed in Supplementary Data 6. Two PVs on a reference genome relative to two draft genomes were defined as shared if at least 75% of their sequences overlap. The *y* axis indicates the number of PVs that are shared under each genome number on *x* axis.

Comparing our predicted two gene sets showed substantial variations in genic regions between the two genomes (Fig. 3a). There are only 10,244 (26.46%) genes in R498 that share identical proteins with 10,289 (27.98%) Nip genes (not a 1:1 relationship). At minimum sequence coverage and identity of both 50%, there are 29,686 (78.68%) genes in R498 and 30,290 (82.36%) genes in Nip having a homologue in its counterpart, and at both 90%, there are only 24,098 (62.24%) and 24,203 (65.81%) homologous genes between the two genomes. We searched the existence of 1,017 known rice gene alleles, including some on the same locus, by aligning their protein sequences to R498 and Nip, and found the majority of them to be different at the protein level between the two genomes and that more than 300 of them are identical to R498 (Supplementary Data 5). There are 66 gene alleles identical to R498 that are different in Nip, including *GW5* (ref. 31), a gene associated with rice grain width and weight, *PSTOL1* (ref. 32) (Fig. 3b), a gene conferring tolerance of phosphorus deficiency, and several blast resistance genes such as *Piz-t* (Fig. 3c), *Pi-km1*, *Pi5-1* and *Pi5-2*. We found more genes in the centromere regions (194 versus 182) and subtelomere regions (437 versus 400) of R498 than Nip (Supplementary Table 9). Moreover, we found that 990 and 966 of our annotated genes were fully contained in the PVs in R498 and Nip, and showed low sequence similarity to the other genome, of which 541 (54.65%) and 381 (39.44%) were annotated with gene ontology (GO) terms, respectively. The enriched R498-specific GO categories include protein phosphorylation, telomere maintenance, transferase activity for transferring phosphorus-containing groups and protein kinase activity (Supplementary Table 12). Of these R498 genes, 605 (61.11%) were found to be expressed in at least one of the eight samples in the R498 RNA-seq data (Supplementary Table 1), and 842 (85.05%) are homologous to other R498 genes.

Although the two genomes contain a similar amount of repetitive sequences, they displayed a significant difference in repeat content, for example, Nip had 17.66% and 4.80% LTR/Gypsy and LTR/Copia elements, respectively, while R498 had 20.55% and 3.86%, respectively (Supplementary Table 8). We found almost six times more solo-LTRs (52,747 versus 7,823) and slightly more full-length LTR elements (7,274 versus 6,686) in R498 than Nip. These results confirmed that rice genomes in two subspecies had experienced independent amplification or loss of LTR retrotransposons after their divergence^16^ (Fig. 3b,c).

### Whole-genome comparison with other rice genomes

We compared 17 deeply sequenced rice draft genomes (including 8 *O. sativa* and 9 *O. rufipogon*) with both R498 and Nip reference genomes to assess the sequence quality of the draft genomes and the diversity of PAVs in rice population (Supplementary Data 6). We found that the R498 sequences were highly similar to the MH63 and ZS97 genomes (Supplementary Fig. 12), with the majority of the known genes in R498 being identical either to MH63 or to ZS97 (Supplementary Data 5), except that the latter two genomes were incomplete^24^. In 93–11, many of the unanchored sequences were aligned to R498 pseudomolecules (Supplementary Fig. 13). We examined the completeness of the 93–11 sequence using the known genes. We found 50 genes, including *WRKY1*, *RPL12-2*, *RCg2* and *RPK*, being partial in 93–11 but with complete sequences in R498, MH63 and ZS97 (Supplementary Data 5), suggesting the incomplete gene assembly in 93–11.

For PAV identification, we focused on only the PVs on the reference genomes, that is, the AVs on the draft genomes, due to the small contig size of most of the draft genomes. We identified from 1,102 to 3,688 PVs on R498 and from 204 to 2,980 PVs on Nip of ≥500 bp relative to the 17 genomes (Supplementary Data 6). As expected, we found fewer PVs between the varieties within subspecies than between subspecies. For example, relative to Koshihikari, a *japonica* variety, only 204 PVs were identified on Nip, but 3,289 PVs were found on R498. Many of these PVs were different from those found between R498 and Nip, and many of them were largely shared by multiple genomes and present in both *O. sativa* and *O. rufipogon* (Fig. 3d and Supplementary Data 7 and 8). On the other hand, the majority (83.48%) of the AVs on each draft genome relative to R498 and Nip were on different positions (Supplementary Data 6). These results indicate that different PAVs are also widely present between rice genomes due to genome-specific gain or loss of DNA fragments, similar to the results previously reported in maize^4^, even though rice has much less genomic diversity than maize at population level.

## Discussion

We here present a highly contiguous and near-complete genome assembly for *indica* rice cultivar R498 through a cost-effective integrated strategy. This assembly contains five centromere gaps and up to 14 potential duplication gaps (Supplementary Table 3), and thus is more continuous than the current reference sequences of Nip, *Arabidopsis thaliana* (https://www.arabidopsis.org, TAIR10 with 96 gaps) and *Brachypodium distachyon* (http://www.brachypodium.org/, v3.1 with 24 gaps) that were obtained by clone-based assembly methods. The high contiguity of the assembly was achieved through combination of linkage map-assisted contig grouping/ordering and fosmid clone-assisted contig connections. The connection process performed the following functions: (1) fixed misordered or unoriented WGS contigs anchored on genetic map, (2) placed unanchored WGS contigs onto the right positions on chromosomes, (3) identified and split chimeric contigs generated in the whole-genome assembly, (4) connected immediately adjacent contigs into super-contigs using fosmid contigs and (5) removed redundancy present in WGS contigs. We used BioNano genome maps to validate the high accuracy of the method and help correct many of the assembly or connection errors. The key to the success of this method is that many globally unassembled repetitive regions (especially segmental duplications) due to conflicts on assembling graphs could be resolved in a local context, either with the help of neighbouring information of contigs on the linkage map, or through the reduced genome complexity in a small portion of the genome (that is, fosmid clones), or by the combination of both.

It is well known that plants have a greater number of complex repetitive sequence structures than animal genomes. The size of plant genomes varies by four orders of magnitude, up to 152 Gb^33^. To resolve the repeat regions presents a major challenge for plant genome assembly. Although the rice genome is small, our whole-genome assembly generated very short contigs (maximum N50 of 1.1 Mb from PBcR) compared with the assemblies of many other species^11^,^27^, suggesting that rice is a highly complex genome, possibly due to its well-preserved ancient whole-genome duplications^34^ or recently amplified retrotransposons^16^. Therefore, the strategies used here should be generally adaptable to the assembly of larger plant or animal genomes. Furthermore, our reported work here can also be viewed as proof-of-concept on how to create high-quality and near-complete platinum genomes, as discussed by Chaisson *et al*.^12^, by leveraging easily accessible technologies. Specifically, the method can be easily extended by replacing genetic map with Hi-C map^35^ or replacing pooled fosmid clones with pools of 10 DNA fragments of >50 kb that are generated by recently developed Chromium Genome system from 10 × Genomics^36^.

This R498 assembly provides an extra resource for gene discovery and for studying genetic variations and genome evolution in rice. We found several genes being present in R498 but missing in Nip and that the majority of the genes have sequence variations between the two genomes. Compared with Nip, the R498 assembly showed superior quality in many repetitive regions. We found one more nucleolus organizer region on chromosomes 10, more genes in the centromere and subtelomere regions and more LTR elements in R498 than in Nip. We assembled a complete R498 mitochondrial genome that was longer and more accurate than that of Nip. We also found that the amount of the organelle DNAs integrated into the rice nuclear genome might be less than previously observed in Nip. The near-complete chromosome-scale R498 genome serves as an ideal reference for linkage-based mutant gene identifications^37^ and genome-wide association studies in *indica* rice subpopulations.

## Methods

### Plant materials and DNA/RNA extraction

*O. sativa* ssp. *indica* (2*n*=2*x*=24) is one of the two major rice subspecies planted in tropical or subtropical regions. The cultivar Shuhui498 (R498) is a major restorer line in a three-line hybrid system planted in Sichuan Province, located in southwest China. We developed a segregating population from a cross between R498 and Nipponbare and planted the population to F_3_ generation. The tissues collected for RNA-seq include: roots (4 weeks), stems (6 weeks), leaves (2 and 6 weeks), spikelets (2–3 days before and 2–3 days after heading) and hulls (7 and 14 days after heading).

For WGS sequencing, high-molecular-weight genomic DNA was extracted from 10-day-old leaves of R498 (multiple seeds) with chloroform–isoamyl alcohol (24:1) followed by cetyltrimethylammonium bromide^38^ extraction and precipitation using 0.7 volumes of isopropanol. Then they were washed by 1 ml 70% ethanol and dissolved in sterile deionized water. RNA from the rice tissues were extracted with TRIzol (Thermo Fisher Scientific, Waltham, MA, USA). Following the cetyltrimethylammonium bromide method, the DNA of the F_3_ population was extracted from the green leaves of individuals planted in the field.

### WGS SMRT sequencing on PacBio platform and *de novo* assembly

The sequencing libraries were prepared with the standard protocol from PacBio and sequenced by Wuhan Nexomics (http: //www.nextomics.cn) on the PacBio long read sequencing instrument, model RSII, with both P5 Polymerase Binding and C3 Chemistry Kits and P6 Polymerase Binding and C4 Chemistry Kits.

The raw PacBio reads were error-corrected and assembled using the PBcR^27^ pipeline with default low stringent parameters: -length 1000 -partitions 300 -sensitive -threads 8 -noclean merSize=14 asmOvlErrorRate=0.10 asmUtgErrorRate=0.07 asmCgwErrorRate=0.10 asmCnsErrorRate=0.10 utgGraphErrorRate=0.07 merylMemory=32000. The high stringent criteria for CA used in the assembly of the whole genome was AS_OVL_ERROR_RATE=0.04 AS_OVERLAP_MIN_LEN=1000. The corrected sequences were 17 Gb, and the longest 10 Gb sequence was selected automatically by PBcR for assembly. The Falcon pipeline (v0.3.0) (https://github.com/PacificBiosciences/FALCON-integrate) was used to do an independent whole genome assembly using the following parameters: length_cutoff_pr=8000 pa_HPCdaligner_option=-v -dal128 -t16 -e.70 -l1000 -s1000 ovlp_HPCdaligner_option=-v -dal128 -t32 -h60 -e.96 -l500 -s1000 pa_DBsplit_option=-x500 -s400 ovlp_DBsplit_option=-x500 -s400 falcon_sense_option=--output_multi --min_idt 0.70 --min_cov 4 --local_match_count_threshold 2 --max_n_read 150 --n_core 6 overlap_filtering_setting=--max_diff 70 --max_cov 300 --min_cov 2 --bestn 15. The CANU (http://canu.readthedocs.org/) pipeline was run with the default parameters. The contaminated sequence contigs from bacteria, fungi or human genome were removed after BWA aligning to the downloaded sequences from GenBank with alignment length >1 kb.

### WGS sequencing on Illumina platform and *de novo* assembly

The sequencing libraries with insert size that peaked at 450 bp were prepared using a PCR-free protocol (Illumina Kit FC-121-3001; Illumina Inc., San Diego, CA, USA). After library profile analysis was performed by an Agilent 2100 Bioanalyser (Agilent Technologies, Santa Clara, CA, USA) and quantitative PCR quantification, the library was sequenced to 2 × 150 bp on the Illumina HiSeq X 10 platform (Illumina).

The short reads were filtered by removing adaptor contaminations and low-quality reads with base N's or more than 10% of bases with a quality score <20. The *de novo* assembly of the WGS short reads was performed by SOAPdenovo (http://soap.genomics.org.cn/SOAPdenovo.html), with kmer=83. The assembled sequences were filtered by removing those aligned to microbial and human genomes (minimum aligned length is 100 bp) downloaded from GenBank with BWA-mem (http://bio-bwa.sourceforge.net/) default parameters.

### RNA sequencing on Illumina platform and data analysis

RNA-seq libraries were prepared with Illumina TruSeq RNA Library Prep Kits v2, with insert size of 300–400 bp and sequenced to 2 × 144 bp on the Illumina HiSeq 2500 platform (Illumina).

RNA-seq short reads were aligned to genome with Hisat2-2.0.1-beta (https://ccb.jhu.edu/software/hisat2/index.shtml), and the expression level of each gene (fragments per kilobase of exon per million fragments mapped) was computed by cufflinks v2.1.1 -u (https://github.com/cole-trapnell-lab/cufflinks). A gene is considered to be expressed if its fragments per kilobase of exon per million fragments mapped>0.

For RNA-seq-based genome coverage computation and SNP calls, only uniquely mapped reads were selected, and SNPs were obtained by GenomeAnalysisTK-2.7-2, UnifiedGenotyper, with filtering criteria of depth ≥5, SNP quality ≥50, cluster SNP within 10 base.

### Genome sequence alignments and SNP calling

We used the BWA-aln and BWA-mem (http://bio-bwa.sourceforge.net/) default setting to align all Illumina short reads to the assembled genome. After alignment, we used SAMtools^39^ to filter out low-quality (mapping quality <30) alignments, and then used the Genome Analysis Toolkit (GATK) (https://www.broadinstitute.org/gatk/) UnifiedGenotypers to call SNPs. The SNPs were filtered using the GATK VariantFiltration program with the following criteria: clusterWindowSize:10, MQ0>=4 && ((MQ0/(1.0 * DP))>0.1), QUAL<50.0, DP<5. The PacBio raw reads were aligned to the genome by BLASR^7^ or BWA.

### Genetic population sequencing and map construction

An F_3_ population of 365 individuals from a cross of R498 and Nip were selected to perform double restriction enzyme (*Bam*HI and *Msp*I) GBS sequencing^26^. DNA samples (∼300 ng per sample) were digested for 2 h at 37 °C with *Msp*I and *Bam*HI (New England BioLabs Inc., Ipswitch, MA, USA) in 15 μl volumes and heated for 20 min at 65 °C. *Msp*I and *Bam*HI adapters were ligated to sticky ends by adding 5 μl of ligation mix containing T4 DNA ligase (NEB) at 22 °C for 2 h and incubated at 65 °C for 20 min to inactive the T4 ligase. Sets of 96 digested DNA samples, each with different barcode adapters were combined (5 μl each) and purified using a commercial kit (QIAquick PCR Purification Kit; Qiagen Inc., Valencia, CA, USA) according to the manufacturer's instructions. DNA samples were eluted in a final volume of 45 μl and those ranged from 300–400 bp were size selected on 2% agarose gel. Amplification of the library was performed using 11 PCR cycles containing an Illumina common adapter primer and an index adapter primer. Sequencing was conducted by using an Illumina HiSeq 2500 instrument (Illumina) to generate 2 × 125 bp reads.

The short reads were aligned to R498 PBcR LS contigs, and SNPs were called as described above. After filtering, we identified a total of 2,087,353 homozygous SNPs between the parents on 1,158 contigs. These SNPs were used to filter out unmatched SNPs or extremely unevenly distributed SNPs (<5% for any one parent genotype) in the F_3_ population. Finally, we obtained 491,128 SNPs on 787 contigs for linkage map construction. Sliding windows of size 50 SNPs were used to create SNP bins to find recombination sites. JoinMap4.1 (https://www.kyazma.nl/index.php/mc.JoinMap/) ML methods were used to cluster the bins into LGs, and then the MstMap (http://alumni.cs.ucr.edu/~yonghui/mstmap.html) Kosambi model was used to compute the order of the bins.

### Fosmid library sequencing and assembly

The R498 fosmid library was constructed by following the protocol of the CopyControl Fosmid Library Kit (Epicentre Biotechnologies, Madison, WI, USA) using high-quality DNA (>200 ng μl^−1^) extracted with a DNeasy Plant Mini Kit (Qiagen) from 2-week-old leaves of R498. The library consisted of 1–2 million independent clones which were stored in four pieces of 384-well plates of 1,000–1,500 clones per sample. The average clone insert size was 36 kb and the whole library was equivalent to about 100–200 × genome coverage. We selected 564 pooled samples for sequencing in six barcoded GBS sequencing libraries, each containing 96 pools.

The fosmid clones were grown in LB broth with 12.5 μg ml^−1^ chloramphenicol in 2-ml-deep 96-well plates shaking at 400 r.p.m. under 37 °C for 10 h and continued for 6 h after adding 0.1 mg ml^−1^ L-(+)-arabinose for inducing multiple copies. The plates were centrifuged for 50 min at 4,000 r.p.m. to collect bacteria, and the fosmid DNA was extracted according to a standard user manual of the plasmid DNA purification NucleoSpin 96 Flash Kit (Macherey-Nagel, Düren, Germany). DNA was finally dissolved in 40 μl of sterile deionized water. We used the same GBS protocol^26^ as used for F_3_ population sequencing to sequence the fosmid DNA library. The libraries were sequenced to 2 × 125 bp on the Illumina HiSeq 2500 platform (Illumina) (a total of 6.3 Gb sequences, 3 × on each tag).

GBS sequence tags in each pool were aligned to the corrected PacBio reads by BWA-mem, and PacBio reads were selected according to the following criteria: up to 20 × with the highest coverage × identity by sequence tags, with alignment identity at least 97%. Only aligned GBS tags in full length were used to compute sequence coverage of PacBIo reads except at the ends of the PacBio reads, where >50% of the GBS tags must be aligned. We assembled these long reads using Falcon with the following parameters: ovlp_HPCdaligner_option=-v -dal4 -t32 -h60 -e.90 -l500 -s1000, overlap_filtering_setting=--max_diff 50 --max_cov 50 --min_cov 1 –bestn 10. All contigs were put together after assembly, and contigs shorter than 10 kb were removed. The fosmid contigs were further assembled into a non-redundant genome with CANU.

### Construction of super-contigs

An overlap graph was constructed using WGS contigs as nodes and fosmid contigs as edges. A WGS contig as node has one or two ends connecting to overlapping fosmid contigs. A fosmid contig as edge has two sequence overlaps on its opposite ends. The score of an edge was defined as:


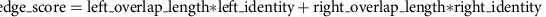


The weighted score for a pair of nodes is the sum of scores of all the edges between them satisfying the predefined thresholds on the overlap identity and length, and the overhang length. The weighted scores for all pairs of connected nodes were recomputed during each step of the connection process using the valid edges. The length of an edge is defined as the sequence length on the fosmid contig between the two overlaps connecting two nodes:


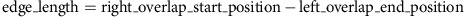


Note that an edge length can be negative if a pair of WGS contigs has sequence overlap between them (Fig. 1e).

The WGS contigs were connected with fosmid contigs to form super-contigs as follows. First overlap graphs were built by aligning all WGS contigs against themselves and aligning all fosmid contigs to all WGS contigs with BWA to find sequence overlaps. This step was repeated for all newly constructed super-contigs as necessary (as sometimes BWA is unable to find all valid overlaps between contigs, the query and target were exchanged and BWA alignment was redone).

Second, nodes were iteratively merged to represent neighbouring contig pairs in a LG with the best-weighted score of all edges with overlap alignment identity >99%, matched length >5 kb and overhang of <1 kb. They were not merged if there were conflicting overlaps (that is, one end of a contig is connected to more than one WGS contigs with the same weighted score). After each node merge, any edges connecting to the already merged contig ends were removed to reduce conflicts for other nodes.

Thirdly, nodes were iteratively merged to represent neighbouring contig pairs using the same criteria as described above, except the identity was reduced to 98%.

Fourth, the previous two steps were repeated while also allowing connections between an anchored contig and an unanchored WGS contig. Each WGS contig was used only once and the shortest edge was selected in case of conflicts. Once an unanchored contig was merged to an anchored one, its connections to the contigs on other LGs were all removed. This step was repeated until no more reliable connections were left and then repeated again with alignment identity further reduced to 97%. This step allowed the extension of the anchored contigs by connecting to the unanchored WGS contigs after all possible neighbouring pairs were connected.

Fifth, chimeric contigs resulting from the original WGS assembly were found and fixed. If one contig or super-contig had sequence overlap to the middle of another contig with overlap length >5 kb, identity >98% and overhang >1 kb, and the overlap was supported by fosmid contigs, then the latter was split and they were connected. The cleaved sequences of >30 kb were used to fill gaps elsewhere.

Sixth, misassembled WGS contigs around gaps were replaced with more reliable WGS contigs and whole connection process was repeated. Unfilled gaps (without reliable overlaps to any WGS or fosmid contigs) outside of the centromere regions after the previous step were likely due to the misassembled WGS contigs around the gaps. These misassembled contigs were replaced with more reliable contigs (for example, those assembled under more stringent criteria) that were matched to the flanking regions for further connection.

Finally, the unanchored WGS contigs not incorporated onto the chromosomes were connected with fosmid contigs wherever possible.

To output the sequences of the super-contigs, the sequences from the WGS contigs were used for the overlapping regions except for edges with negative length where the sequences from the fosmid contigs were used for the overlapping regions due to the low sequence quality in the overlapping regions of many WGS contig pairs.

### BioNano data generation and analysis

R498 leaves from 10-day-old seedlings growing in dark conditions were collected. High-molecular-weight DNA extraction and DNA labelling (with Nt.BspQI nicking endonuclease) were both performed according the standard protocols provided by BioNano Genomics. Labelled DNA samples were loaded and run on the Irys system (BioNano Genomics) (service provided by BGI http://www.bgitechsolutions.com/index.html). Single-molecule maps of >100 kb were collected.

The single-molecule maps were corrected by the IrysView (v2.5.1) software (BioNano Genomics) with parameter ‘-autonoise', which used the PBcR LS contigs to obtain noise parameters for molecule mapping and assembly. The *de novo* assembly, hybrid assembly and detection of SVs were all done using the same Irys assembler and aligner, with auto selected parameters, and a minimum length of 150 kb. The SVs were stored in a file named *.InDel that was output from IrysView compare module. For hybrid assembly with module HybridScaffold, the sequence contigs were split wherever conflicts were found between them and the genome maps.

### Error correction and assembly of two organelle DNAs

To correct assembly errors in the indels >10 kb identified with genome maps, two WGS contigs of >100 kb around each indel were selected as starting and ending nodes on the overlap graph to enumerate the best-scored paths between them (Supplementary Fig. 5). In case that more than one valid aligned region was found between a WGS contig and a fosmid contig, a link was added to the overlap graph for each aligned region. The path matching to the genome map with the same length and nicking sites was selected as the replacing path. For local reassembly, the corrected PacBio sequences were aligned by BWA-mem to the misassembled regions with indels <10 kb (extending 30 kb to each side of the indels) in the genome or the misassembled cpDNA and mtDNA super-contigs. The sequences with alignment identity >98% and matching length >3 kb were selected for CANU reassembly with default parameters. The reconnected or reassembled sequences were used to replace the corresponding erroneous regions in the pseudomolecules.

Two small super-contigs of 593 and 619 kb after connection were found to be homologous to Nip cpDNA and mtDNA, respectively. After reassembly, the largest cpDNA contig contained a circular DNA of 134 kb. The reassembled mtDNA contigs were connected by fosmid contigs with alignment identity >99% and overlap length >10 kb into a contig of 414 kb. The contig ends were extended with the corrected PacBio reads and reassembled for several rounds, and again were connected with the fosmid contigs until a circular DNA of 527 kb was obtained. The raw SMRT reads were aligned back by BLASR to mtDNA to assure the complete coverage and lack of breaking points as shown in Supplementary Fig. 8.

### Centromere and telomere sequence identification

Centromere-specific (the RCS2 family^40^) repeats were aligned by BWA-mem (default parameters) to the final R498 genome and the assembled contigs to identify centromere-related sequences. The centromere repeat regions in R498 were defined as the regions that contained all full units of the RCS2 family. The telomere sequence 5′-AAACCCT-3′ and all different orders of the seven bases were searched directly.

### Postassembly examination of WGS contigs

All initially assembled WGS contigs were aligned to R498 genome using BWA-mem. Alignment identity ≥97% and coverage ≥95% were used to identify contigs that were incorporated into the final genome assembly. Contaminated microbial DNA was identified with BWA alignment coverage ≥95%. R498 cpDNA and mtDNA were used to identify chloroplast and mitochondrial contigs with BWA alignment identity ≥95% and coverage ≥95%. Centromere-related contigs were identified with BWA alignment coverage ≥95%. The rest of the contigs were designated as unclassified. For computing genome coverage, one contig was allowed to match to only one best-matched genome location. An erroneous contig was defined if it was aligned to one block with overhang >1 kb (>100 bp for short read assembly) or aligned to two or more non-overlapping blocks. For short read assembly, the contigs were filtered by aligning them to microbial and human DNAs with BWA-mem and then mapped to the R498 assembly including cpDNA and mtDNA with BLAT default parameters. The unmapped contigs were aligned to themselves with BLAT to remove those redundant sequences that were covered on >90% of their length by a longer sequence.

### Gene and repeat annotations

The Nip and R498 genes were first predicted by an evidence-based pipeline^29^, using rice and monocot cDNAs/ESTs downloaded from GenBank, proteins downloaded from SwissProt, and rice RNA-seq data that were downloaded from GenBank short read archive (http://www.ncbi.nlm.nih.gov/Traces/sra/sra.cgi) and assembled by SOAPdenovo-trans (http://soap.genomics.org.cn/SOAPdenovo-Trans.html) as evidence. We annotated motifs and domains using InterProScan (http://www.ebi.ac.uk/Tools/pfa/iprscan5/). Gene functions were further annotated according to the best-matched proteins in *Brachypodium distachyon* (http://genome.jgi.doe.gov) or maize (ftp://ftp.ensemblgenomes.org) databases using blastp with 30% minimal identity and coverage. To make reliable comparison of the genes between the two genomes, we created a core set of protein-coding genes by removing low-confidence genes as follows: (1) sharing homology to transposons in plants by more than 100 amino acids or 50% in DNA length, (2) EST only single-exon genes with protein length <50 amino acids or covering <70% of another homologous genes in the former's full protein length (as pseudogenes). The coordinates of the genes in MSU7 (38,869 valid genes) and RAP (35,472 valid genes) were compared to our annotated Nip core gene set to find overlapping genes among them. The comparison of genes between R498 and Nip or within R498 was conducted by protein BLAST (ncbi-blast-2.2.28+ -evalue 1e-5) using the longest protein in each gene. The sequence identity and coverage were computed based on the aligned length and total length of each gene.

The repetitive sequences in both R498 and Nip genomes were annotated by combining *ab initio* and homology-based methods. First, an *ab initio* repeat library was predicted for each genome with LTR_FINDER (http://tlife.fudan.edu.cn/ltr_finder/); the type of each repeat family was assigned by homology to PGSB Repeat Element Database (http://pgsb.helmholtz-muenchen.de/plant/recat/). Second, this library was combined with Repbase (http://www.girinst.org/repbase) for identifying all homologous repeats throughout the genome by RepeatMasker (http://www.repeatmasker.org/) with WU-BLASTX as search engine. Third, since many of the identified repeats overlapped with each other, the overlapping repeats in the same class were combined together. Among the overlapping repeats belonging to different classes, the longer ones were split into pieces at the overlap boundaries and the redundant parts were discarded to ensure only one class assignment for each repeated sequence. Fourth, the tandem repeats were further identified with Tandem Repeats Finder (http://tandem.bu.edu/trf/trf.html). The solo-LTRs and intact LTRs were further classified with LTRharvest (https://omictools.com/ltrharvest-tool).

### Genome comparison and SV identification

Dot plot alignment was conducted with MUMmer^41^ with -mincluster 500. Synteny analysis was also conducted using SyMAP v4.2 (http://www.agcol.arizona.edu/software/symap). The genome coverage of short reads was computed by BedTools coverage (http://bedtools.readthedocs.org). We identify SNPs and indels <500 bp between R498 and Nip genomes using MUMmer with the following four parameters: (1) nucmer -maxmatch -c 90 -l 40, (2) delta-filter -1, (3) show-snps and (4) show-diff. In addition, we used the collinear, non-overlapping, best-matched blocks of minimum 200 bp generated from MUMmer to identify unaligned sequence gaps ≥100 bp between R498 and Nip, and between each draft genome and one of the two reference genomes. The unaligned gap length between R498 and Nip was recomputed by removing the sequences of ≥200 bp with homology to the other genome. The recomputed gap length was treated as the PAV length. For the unaligned gap sequences in a reference genome relative to the draft genomes, we aligned their whole-genome short reads to the reference genome and kept only those ≥500 bp with sequence coverage of <25% as PVs. Two PVs on a reference genome relative to two draft genomes were considered to be shared if they overlapped with each other by >75% of their length.

The genes fully enclosed in the PVs between R498 and Nip were filtered by removing those with matching sequences of ≥200 bp to the other genome; that is, only those genes without significant homology to the other genome were retained for further analysis. For GO enrichment analysis, we used the whole gene set in each genome as background to do Fisher test on the PV-enclosed genes.

### Data availability

All analyses and quality control steps were coded in Perl scripts or Linux shell commands/scripts except where stated explicitly. The custom Perl codes for building overlap graphs and generating final sequences are provided as Supplementary Software. The sequence reads are available at The Genome Sequence Archive (GSA) (http://gsa.big.ac.cn/index.jsp) under project PRJCA000313. The genome assembly of R498, and the data from WGS sequencing, population sequencing and fosmid sequencing have been deposited under NCBI BioProjects PRJNA318714, PRJNA318714, PRJNA318826 and PRJNA340081. The accessions to R498 assembly are CP018157–CP018170, and the accessions to genome sequencing data are SRP073415 and SRP073521. The R498 genome assembly and the annotated genes for both R498 and Nipponbare are also accessible at http://www.mbkbase.org/R498.

## Additional information

**How to cite this article:** Du, H. *et al*. Sequencing and *de novo* assembly of a near complete *indica* rice genome. *Nat. Commun.* **8**, 15324 doi: 10.1038/ncomms15324 (2017).

**Publisher's note:** Springer Nature remains neutral with regard to jurisdictional claims in published maps and institutional affiliations.

## Supplementary Material

Supplementary Information Supplementary Figures, Supplementary Tables, Supplementary Notes and Supplementary References

Supplementary Data 1 Summary of the assembled genome maps.

Supplementary Data 2 Alignment of the genome maps to the final pseudomolecules.

Supplementary Data 3 cpDNAs and mtDNAs in R498 and Nip.

Supplementary Data 4 Genome positions of PAVs (=100 bp) between R498 and Nip.

Supplementary Data 5 Alignment of known genes to R498, Nip, MH63, ZH97, and 93-11.

Supplementary Data 6 Statistics of PVs identified in R498 and Nip by comparing to other rice genomes.

Supplementary Data 7 PVs identified in R498 by comparing to other rice genomes.

Supplementary Data 8 PVs identified in Nip by comparing to other rice genomes.

Supplementary Software The custom Perl codes for building overlap graphs and generating final sequences.

## Figures and Tables

**Table 1:** Comparison of basic sequence statistics of R498 and Nipponbare MSU7.

**Chr**	**R498 Len**	**Nip Len**	R498 Gaps*	**Nip Gaps**†	**R498 Tel**	**Nip Tel**
Chr1	44,361,539	43,270,923	1	12	Both	Single
Chr2	37,764,328	35,937,250	1	6	Both	Both
Chr3	39,691,490	36,413,819	1	12	Both	Both
Chr4	35,849,732	35,502,694	0	46	Both	Both
Chr5	31,237,231	29,958,434	0	14	Both	Single
Chr6	32,465,040	31,248,787	0	5	Both	Single
Chr7	30,277,827	29,697,621	1	9	Both	Both
Chr8	29,952,003	28,443,022	0	3	Both	Single
Chr9	24,760,661	23,012,720	0	18	Both	None
Chr10	25,582,588	23,207,287	0	28	Both	Single
Chr11	31,778,392	29,021,106	1	69	Both	None
Chr12	26,601,357	27,531,856	0	17	Both	None
mtDNA	527,116	490,520	0	0	—	—
cpDNA	134,546	134,525	0	0	—	—
Total**	390,983,850	373,870,564	5	239	24	13

Chr, chromosome; Len, length; Nip, Nipponbare; Tel, telomere.

**R498 GC content, 43.57%; Nip GC content, 43.53%.

^*^R498 centromere gap locations: chr1, 17,339,881–17,349,880; chr2, 13,684,078–13,694,077; chr3, 21,193,261–21,203,260; chr7, 12,695,696–12,705,695; chr11, 13,162,594–13,172,593. More potential duplication gaps are listed in Supplementary Table 3.

^†^The gaps in Nip are those at least 100 bp.

**Table 2:** Comparison of genome features between R498 and Nipponbare MSU7.

**Feature type**	**In R498**	**In Nip**	
*Gene*			
Number	38,714	36,775	
*Repeat content*			
%	42.05	40.43	
*SNP*			
Number	2,548,071	2,548,071	
*Insertion of 1–99 bp*			
Number	226,771	239,390	
Base	1,034,898	1,077,080	
*PV of 100–499 bp*			
Number	3,432	3,411	
Base	887,602	856,902	
*PV of 500–999 bp*			
Number	1,301	1,328	
Base	938,805	962,372	
*PV of ≥1–10 kb*			
Number	5,407	4,973	
Base	19,316,383	16,710,125	
*PV of ≥10–50 kb*			
Number	1,524	726	
Base	28,857,940	13,663,983	
*PV of ≥50 kb*			
Number	170	92	
Base	16,851,530	7,960,212	
*PV of ≥500 bp*			
Number	8,402	7,119	
Base	65,964,658	39,296,692	

AV, absence variation; PV, presence variation; SNP, single-nucleotide polymorphism.

An insertion in R498 is equivalent to a deletion in Nip, and *vice versa*. A PV in R498 is equivalent to an AV in Nip, and *vice versa*.
