# Supplementary material for: Sequencing and de novo assembly of a near complete indica rice genome
Source: Nat Commun. 2017 May 4;8:15324. doi: 10.1038/ncomms15324 (PMC5418594; doi:10.1038/ncomms15324)
Supplement: Supplementary Information — Supplementary Figures, Supplementary Tables, Supplementary Notes and Supplementary References [file ncomms15324-s1.pdf]

Supplementary Figures

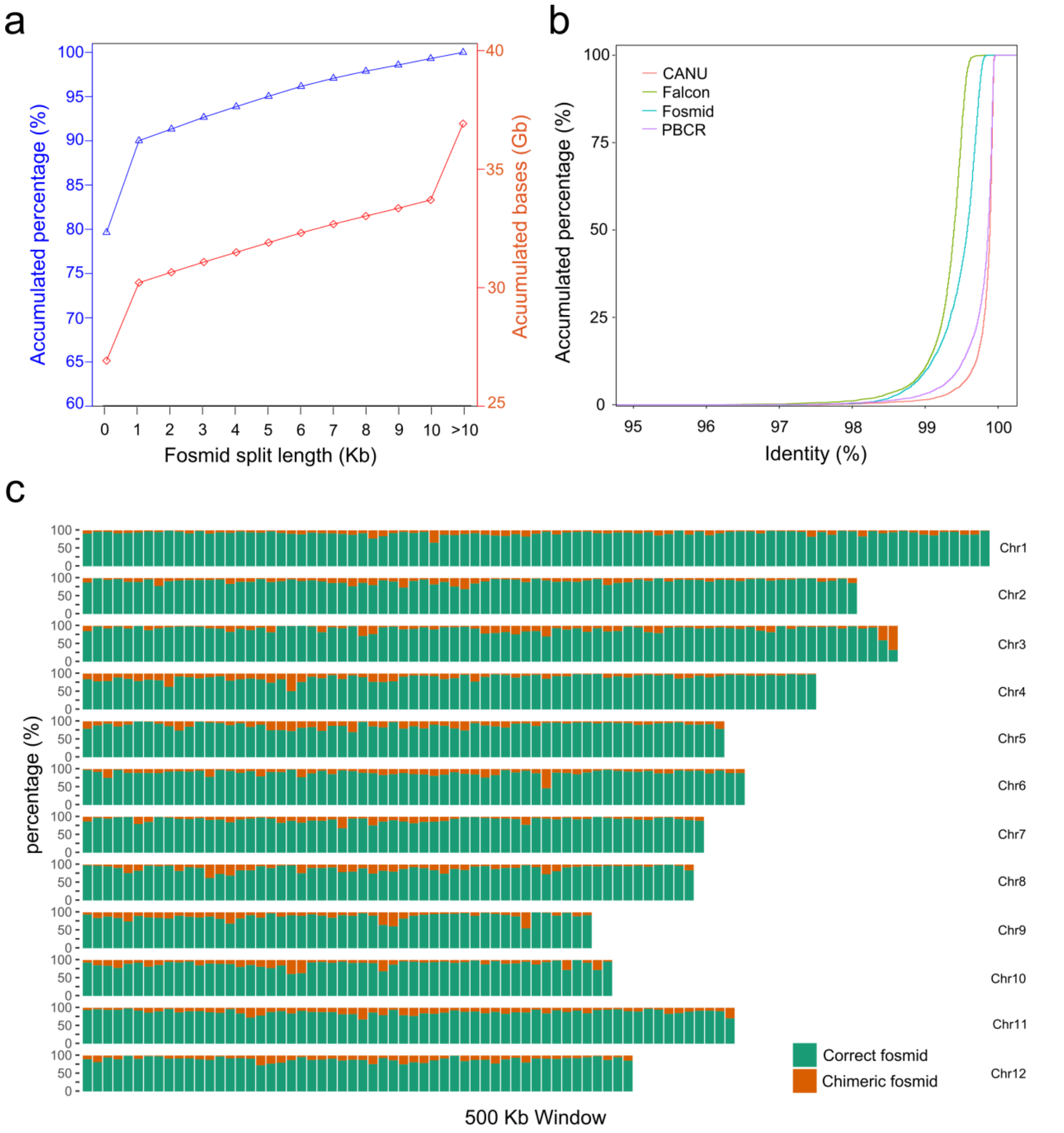

Supplementary Figure 1: Quality assessment of the fosmid contigs. All the fosmid contigs were aligned to the assembled genome by BWA with minimum sequence alignment identity of 97%. (**a**) The x-axis indicates the overhang length (chimeric portion) of a fosmid. The left y-axis indicates the number of contigs under each overhang threshold; the right y-axis indicates the accumulated length of the fosmid contigs under each overhang threshold. More than 95% of the aligned fosmid contigs (>80x genome coverage) have overhang length <5 kb, which is below the minimum overlap length used for connections (ie, not leading to chimeric connections). (**b**) Comparison of the base accuracy distribution of fosmid contigs to that of WGS contigs with the pseudomolecules as reference. (**c**) Distribution of the correct and chimeric fosmid contigs throughout the genome. Each vertical bar represents a 500 kb window.

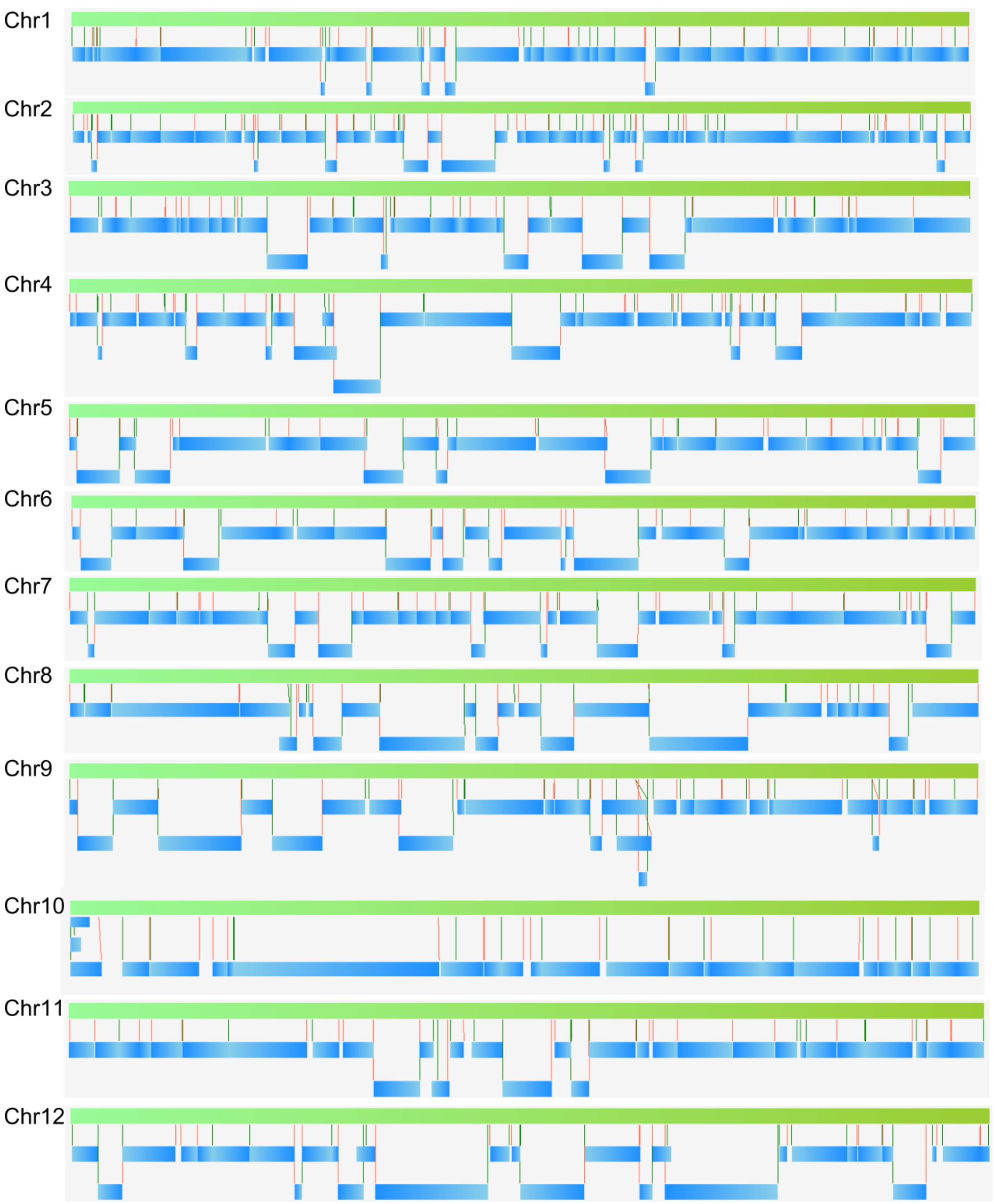

Supplementary Figure 2: Overview of the comparison of all R498 pseudomolecules to genome maps. Each green horizontal bar represents an assembled pseudomolecule. Each blue horizontal bar represents a genome map. The vertical lines represent their matching boundaries. Multiple genome maps assembled from tandem repeats were aligned to the middle of chromosome 9 and the beginning of chromosome 10.

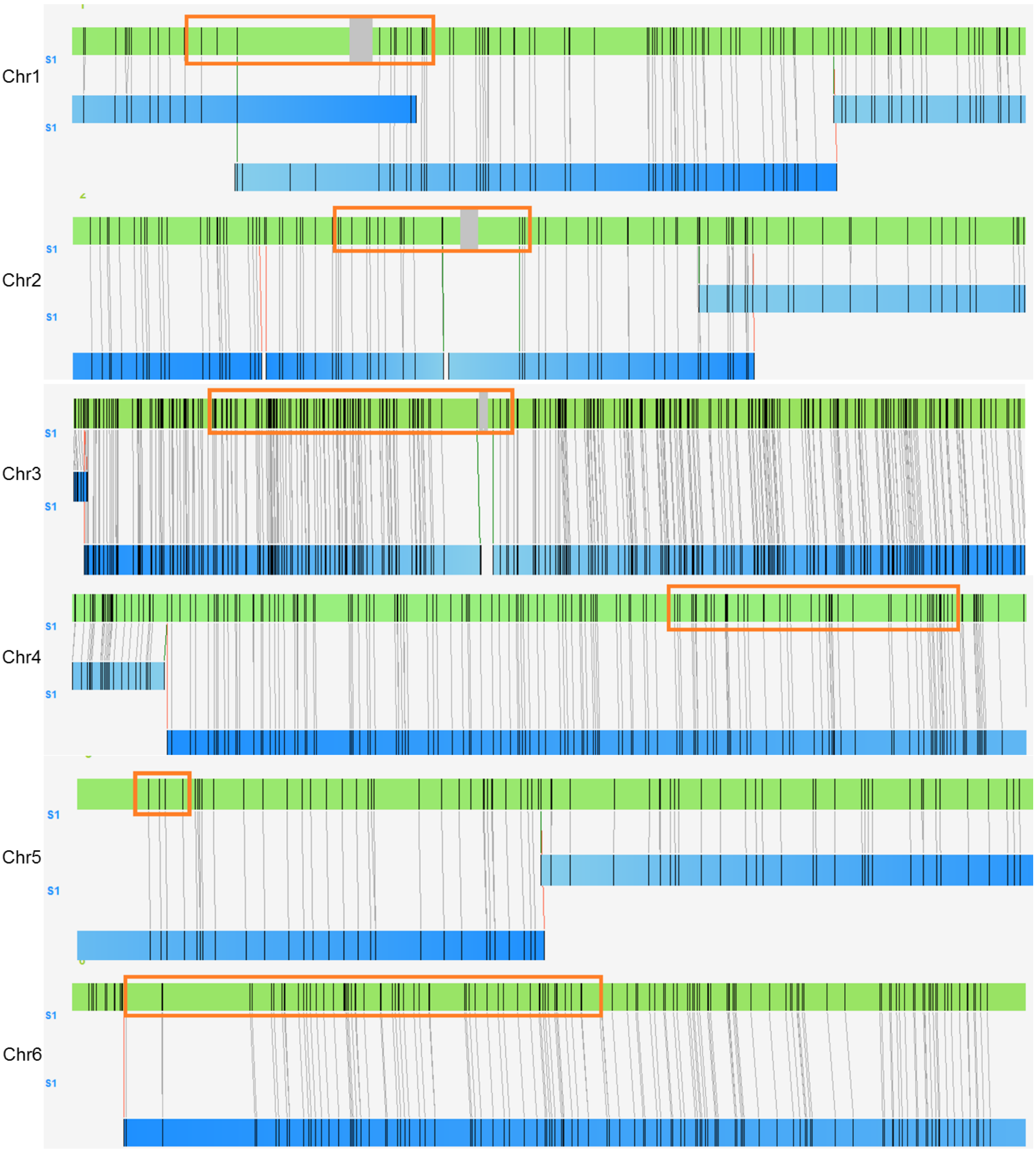
Chr1
Chr2
Chr3
Chr4
Chr5
Chr6

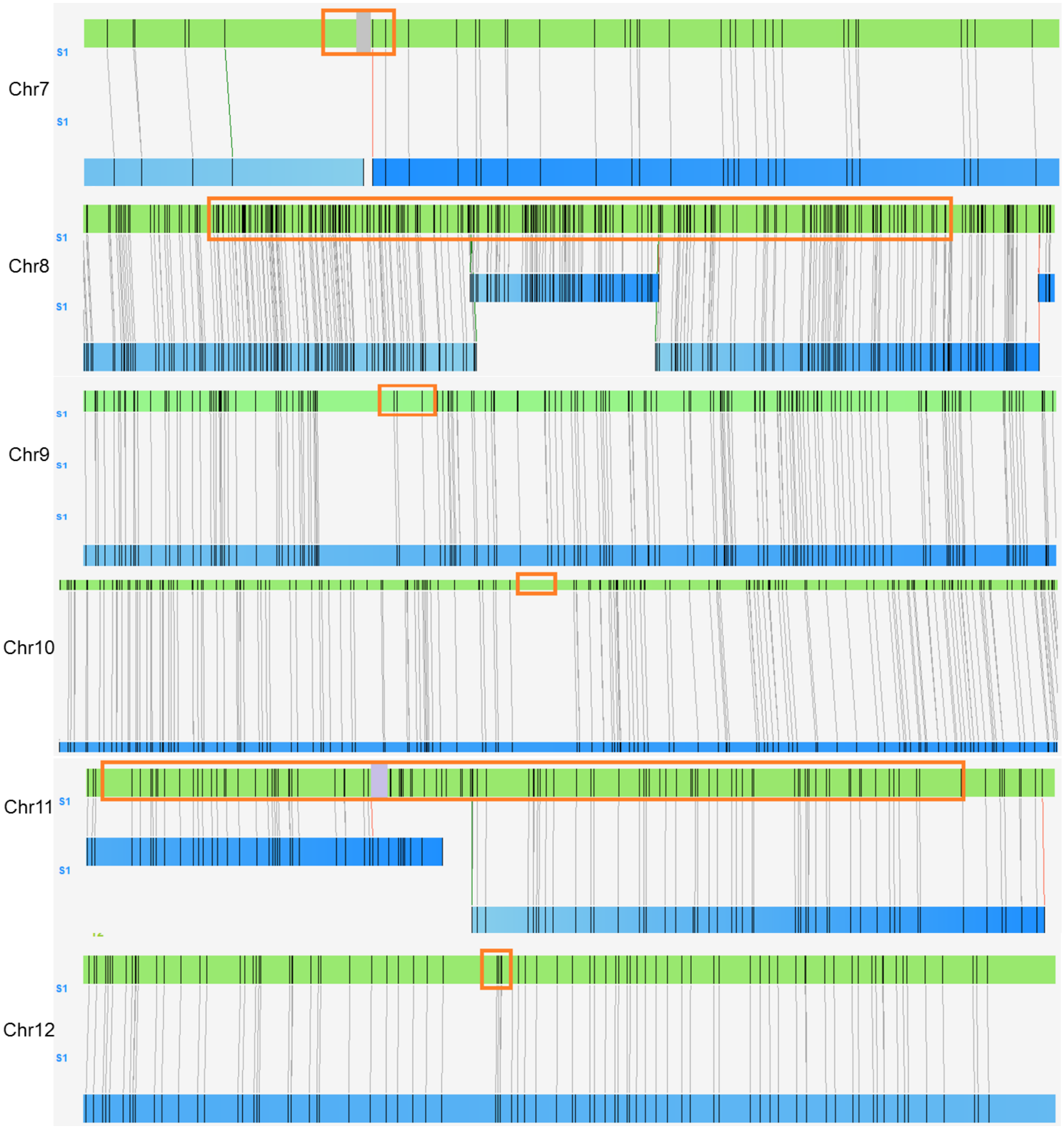

Supplementary Figure 3: Comparison of all the assembled R498 centromere regions with genome maps. The green horizontal bars represent pseudomolecules. The blue horizontal bars represent genome maps. The vertical lines represent their matching nicking sites. Red-lined rectangles represent centromere regions (containing all the full units of rice centromere tandem repeats RCS2; see Supplementary Table 9 for their starting and ending positions), and gray boxes represent gaps. None of the five gaps was completely covered by a single map. Therefore, their sizes could not be estimated.

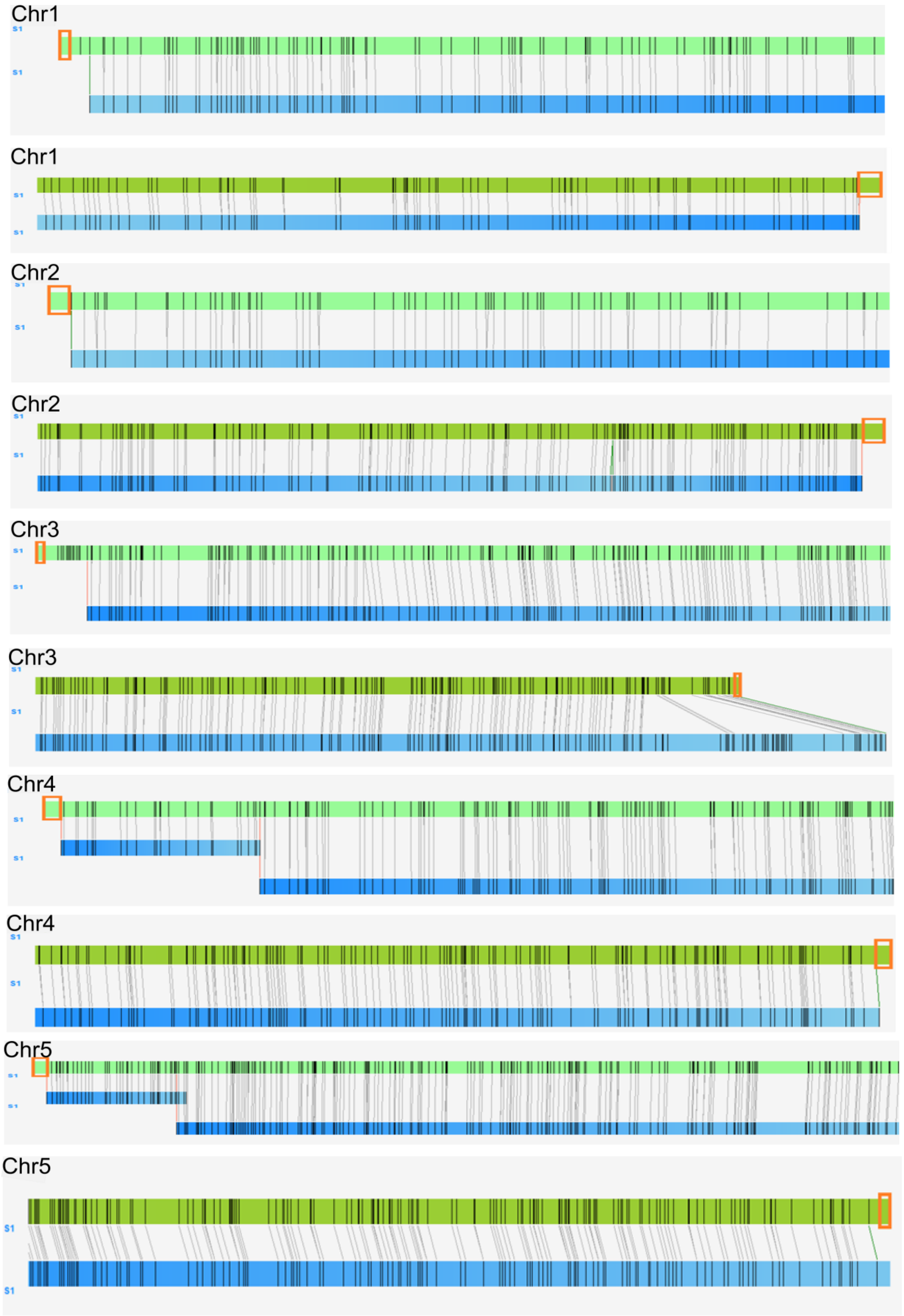
Chr1
Chr1
Chr2
Chr2
Chr3
Chr3
Chr4
Chr4
Chr5
Chr5

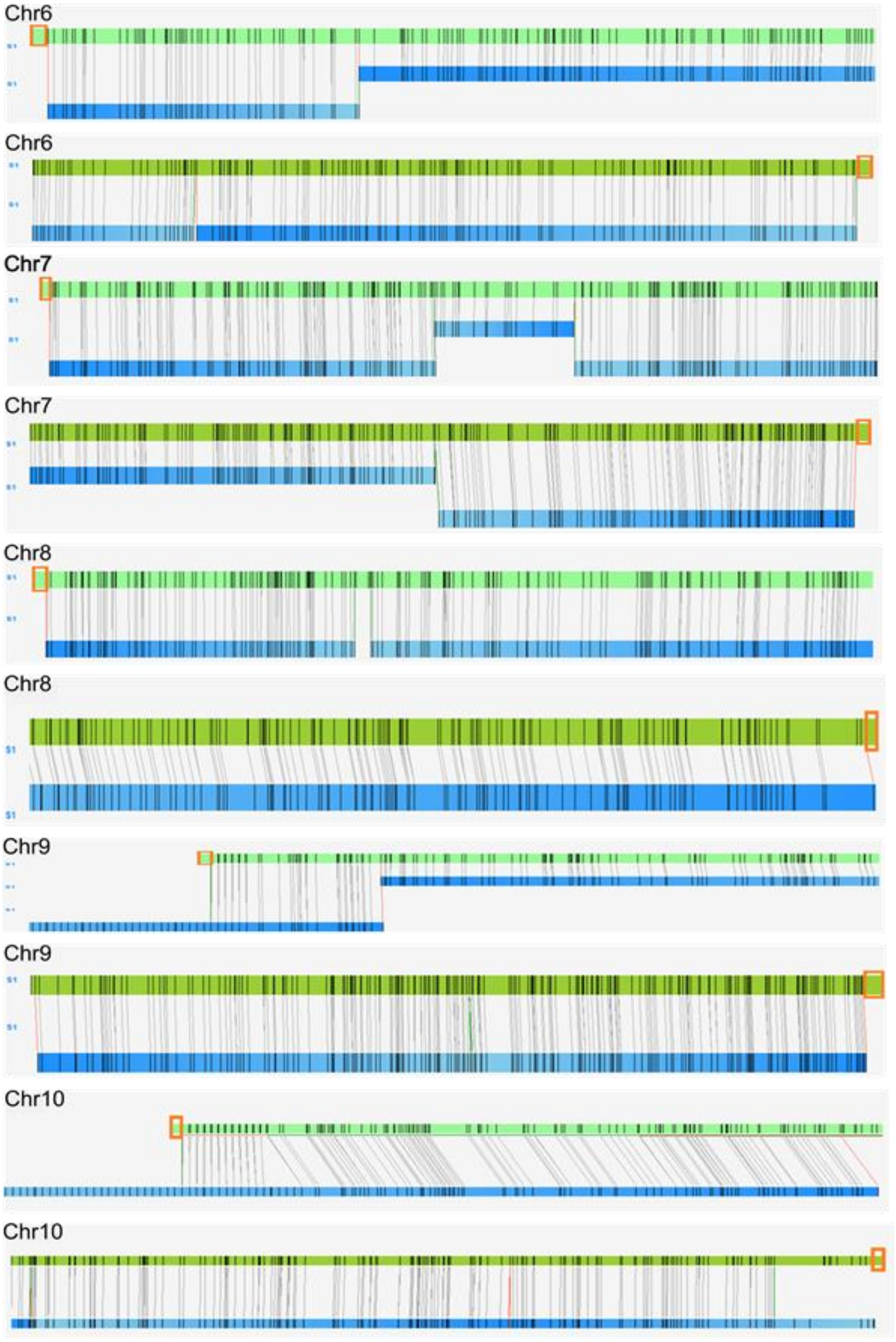
Chr6
Chr6
Chr7
Chr7
Chr8
Chr8
Chr9
Chr9
Chr10
Chr10

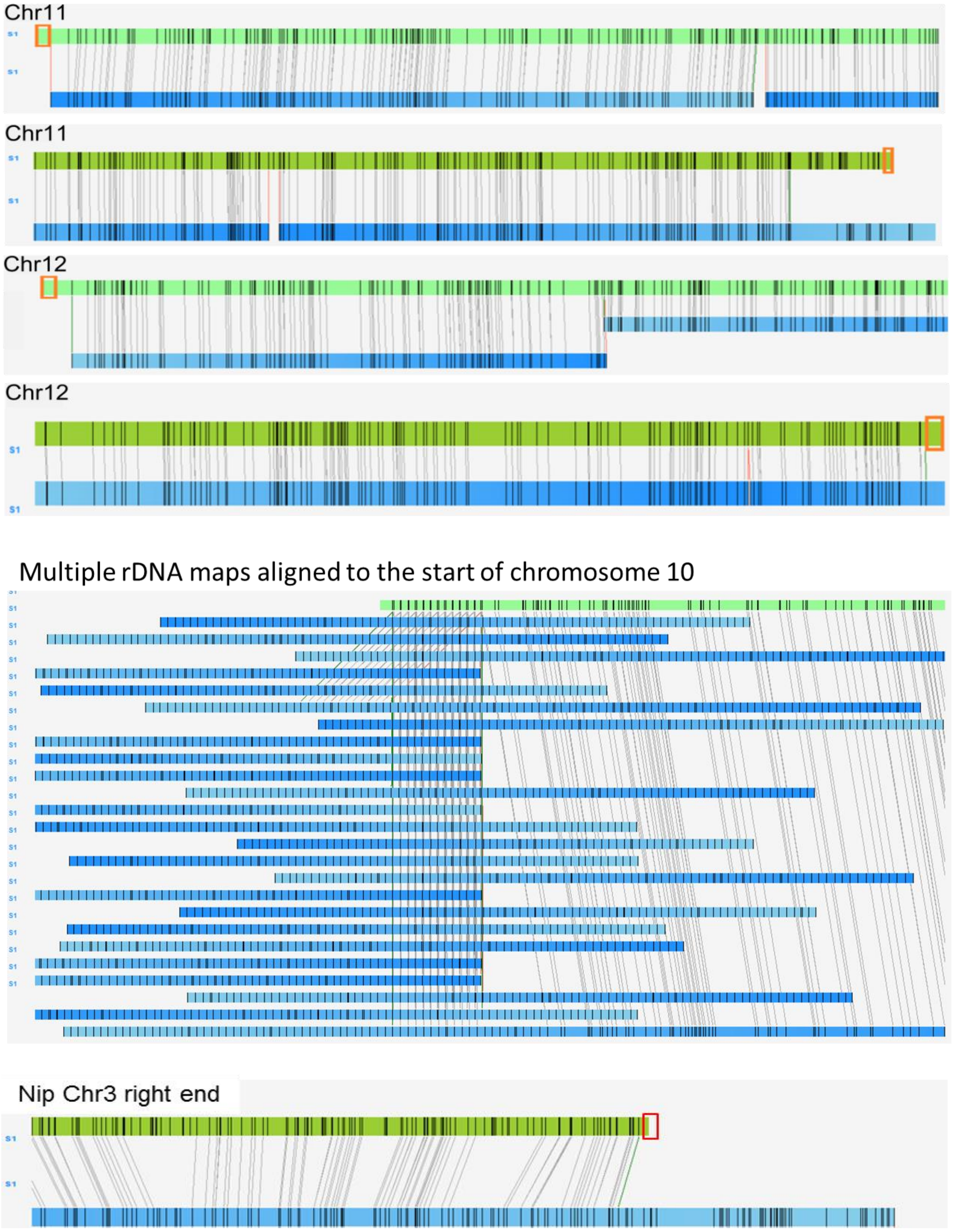

Supplementary Figure 4: Comparison of R498 chromosomal ends and Nip chromosome 3 distal ends with R498 genome maps. Red-lined rectangles indicate telomere repeats. The rDNAs are located at the beginning of the chromosomes 9 and 10 (multiple rDNA maps are aligned to chromosome 10). The comparison of R498 genome map to the distal end of chromosome 3 in Nip shows the incomplete end of Nip chromosome 3, missing the corresponding region in R498 which includes the two deletions.

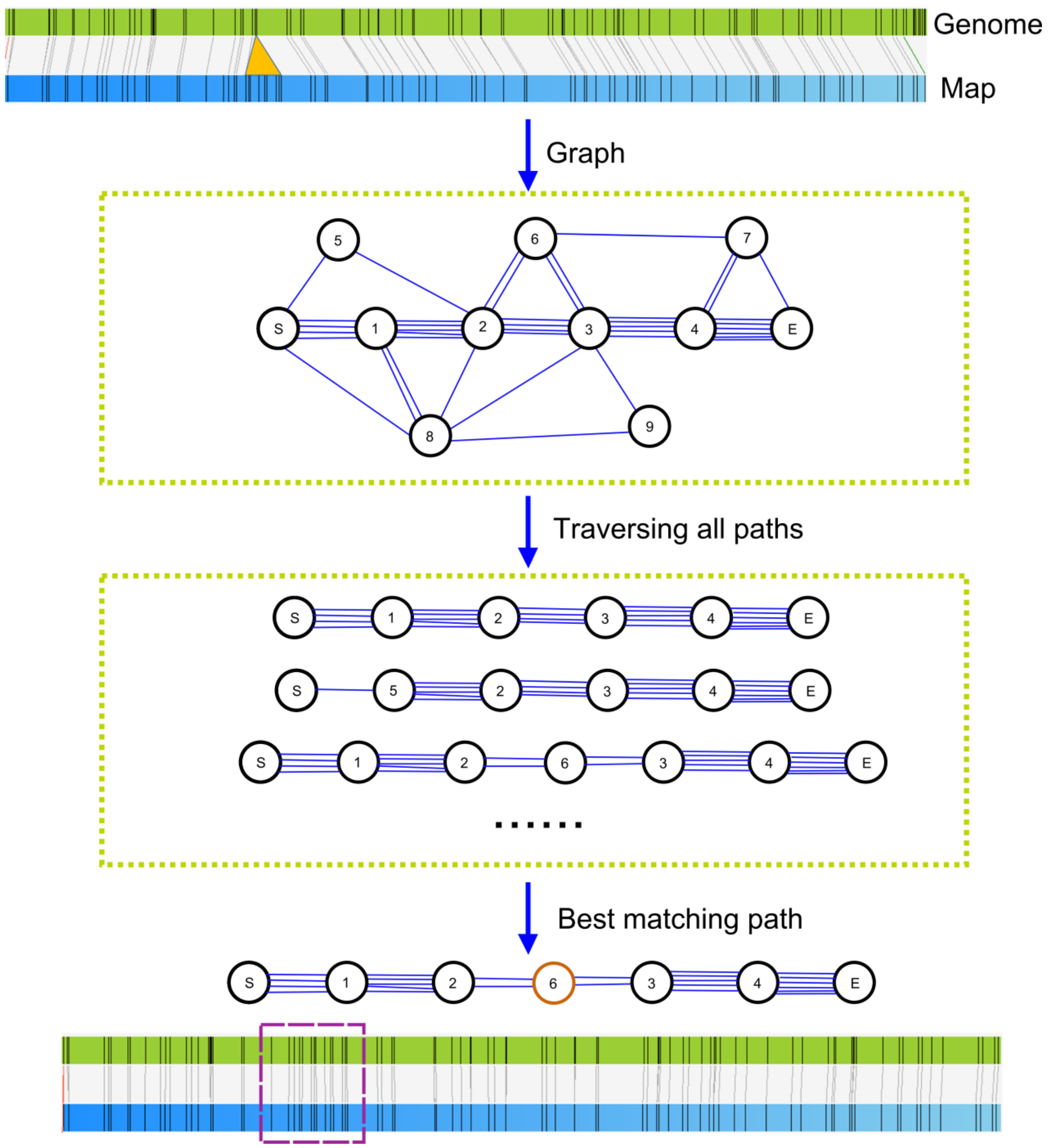

Supplementary Figure 5: Correction of indels in the super-contigs. A deletion was found in the assembled sequences (orange on top). The region on overlap graph is shown in the middle. The blue lines represent fosmid contigs and the numbered circles represent WGS contigs. This overlap graph contains many alternative paths in a repetitive region, of which a wrong path was selected initially. Under the guidance of genome maps a new path with the correct length and corresponding nicking sites is selected to fix the error. S and E represent the starting and ending WGS contigs $\geqslant$ 100 kb that are nearest to the indel in the region, respectively. The purple dashed box at the bottom represents the region after error correction with matching length and nicking sites. See Supplementary Table 13 for a list of corrected regions with changed WGS contigs.

A

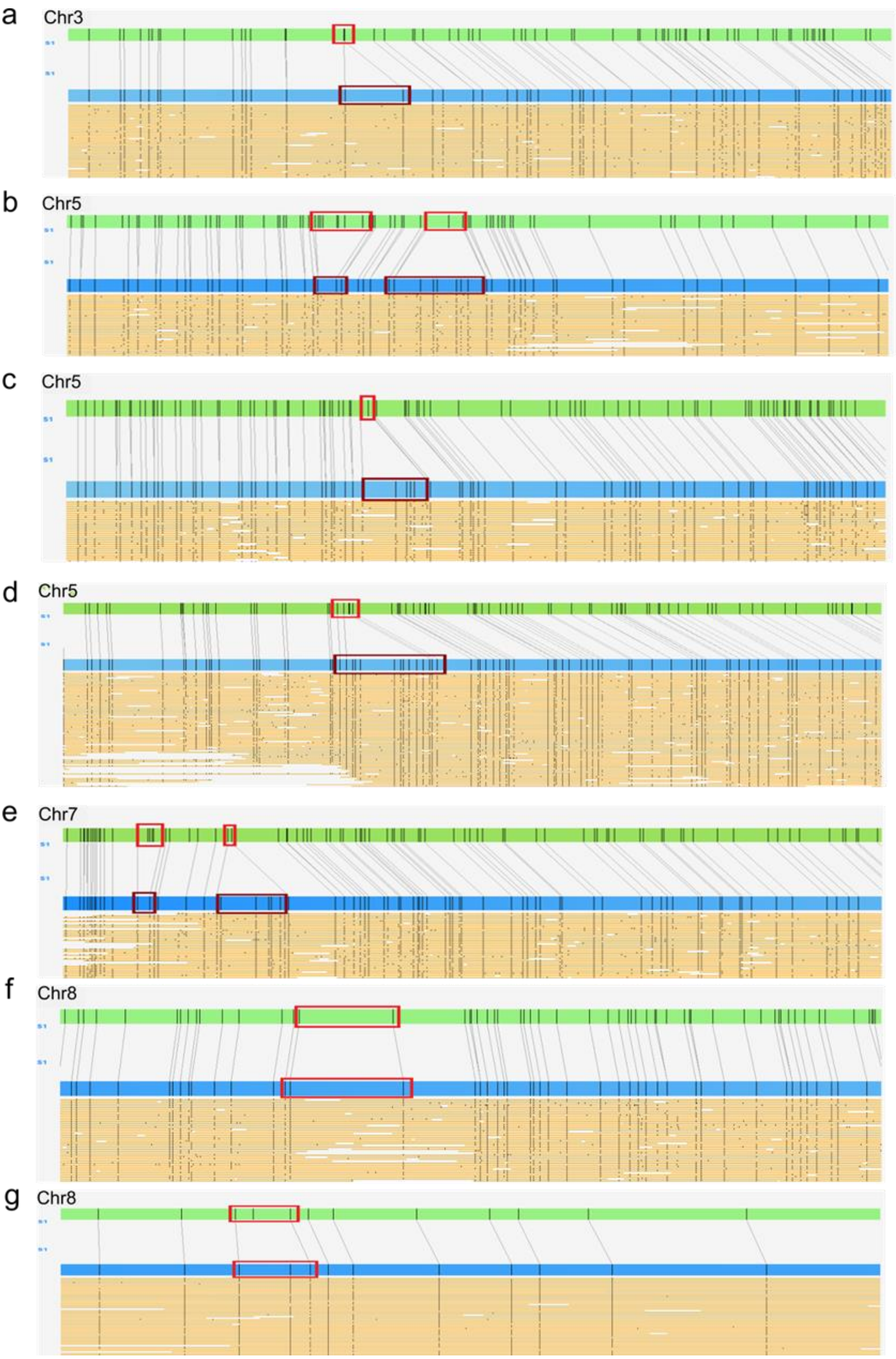

A (continue)

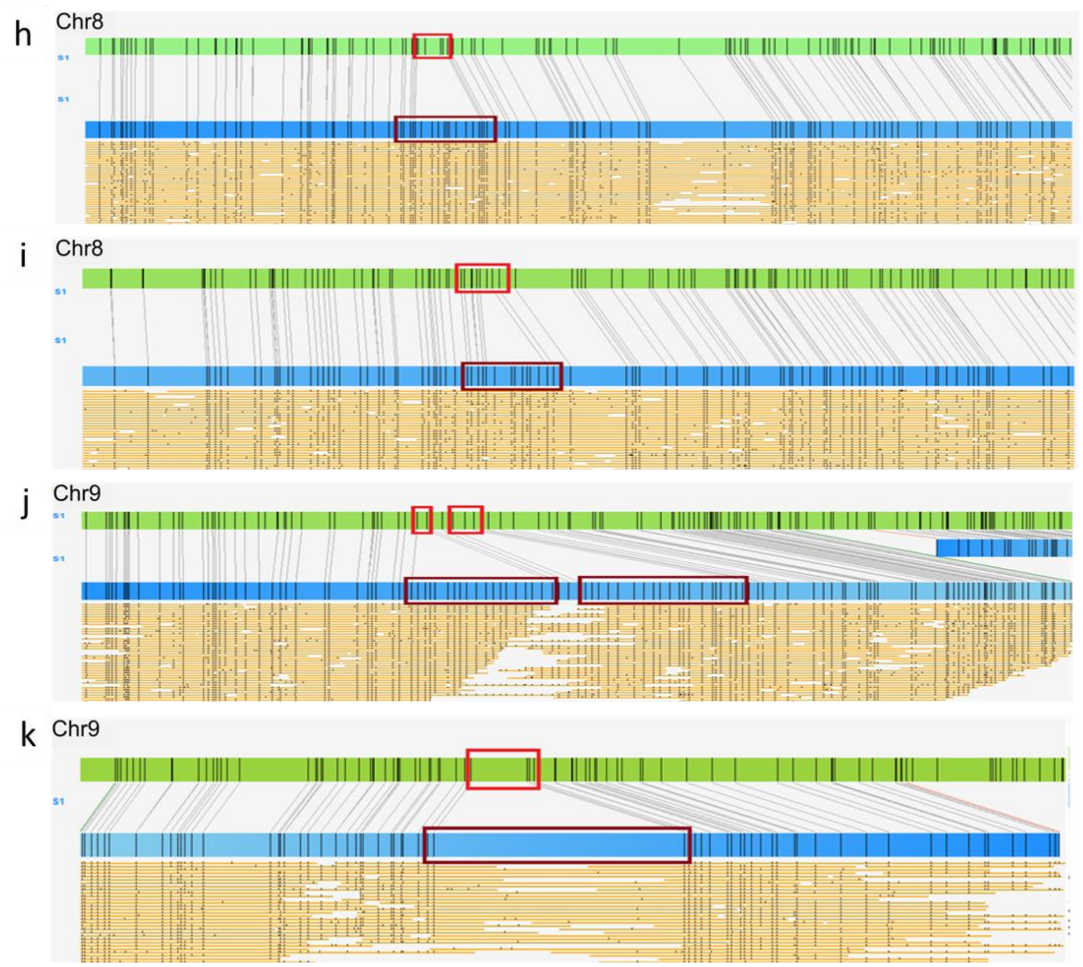

B

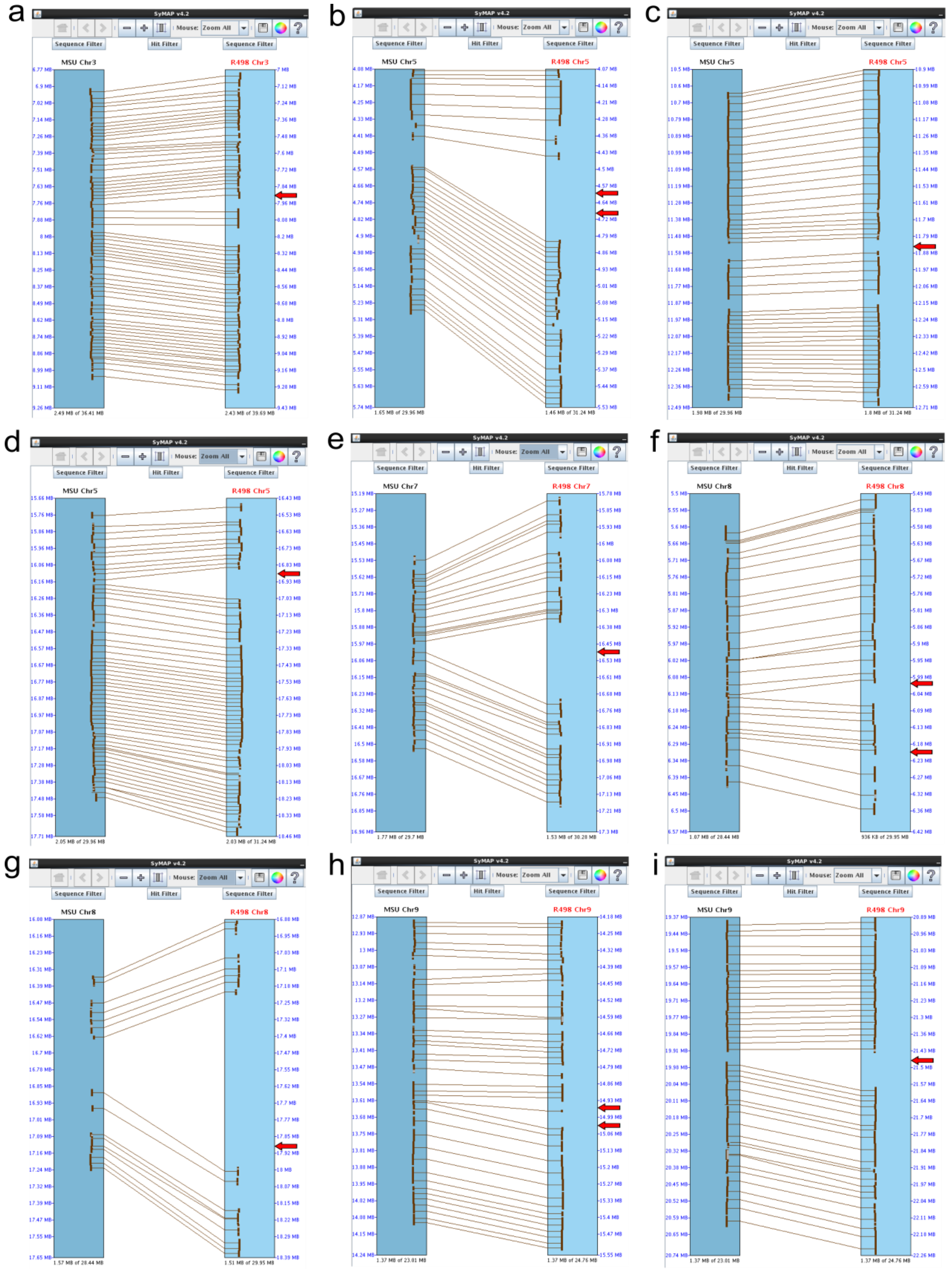

Supplementary Figure 6: The potential error-prone regions compared to genome maps. See Supplementary Table 3 for their positions on pseudomolecules. (**A**) Comparison of R498 pseudomolecules to genome maps to show indels. (**B**) Synteny between R498 and Nip in the regions around the indels in (**A**). Red arrows indicate the location of each indel.

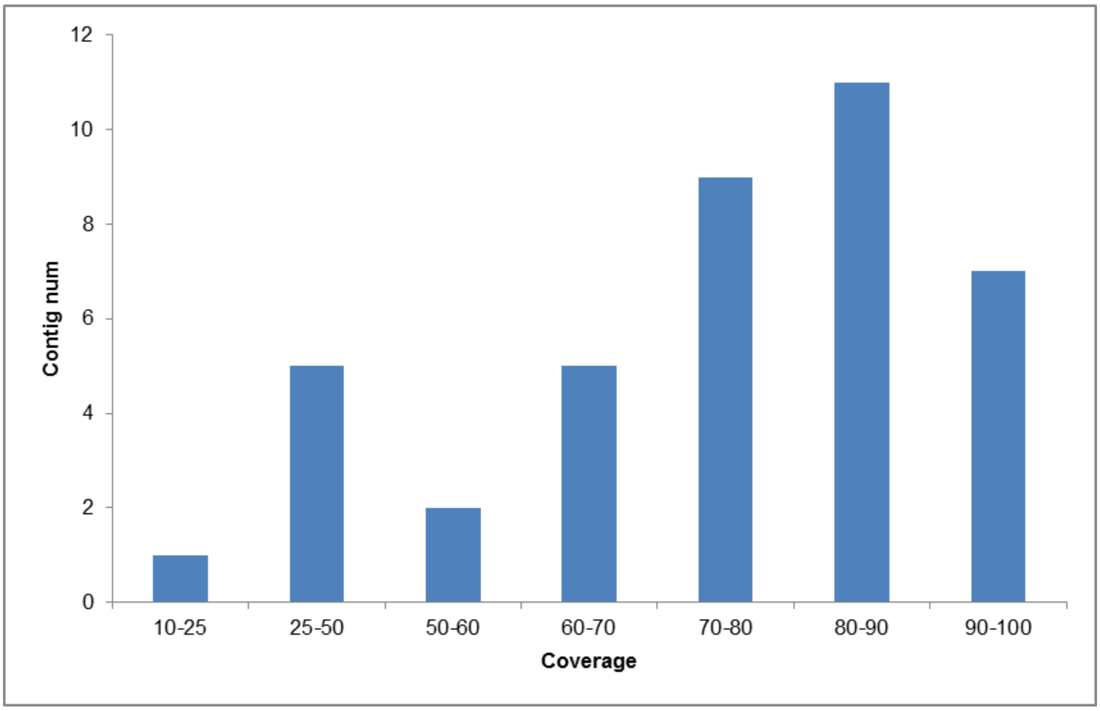

Supplementary Figure 7: Quality assessment of the unclassified PBcR HS contigs using Illumina short reads. The x-axis indicates the sequence mapping coverage (%) of the contigs by Illumina short reads. The unclassified contigs have much lower sequence coverage than the pseudomolecules (99.94%) by Illumina short reads. Aligned SMRT reads to these contigs were retrieved and assembled into 419.8 kb of sequences. Of them 415.1 kb (98.88%) were aligned to the assembled R498, including a cpDNA contig of 159.6 kb and an mtDNA contig of 43.7 kb.

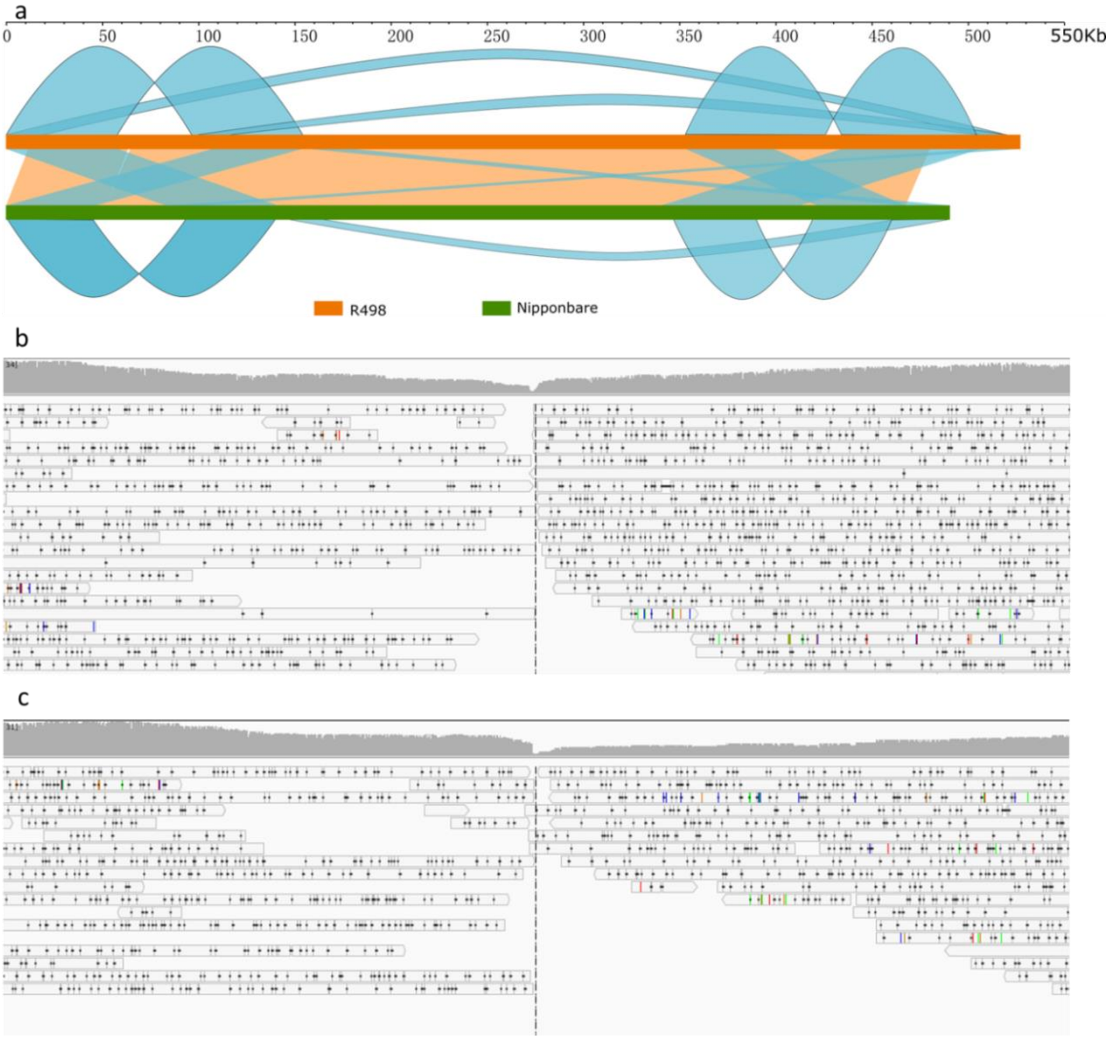

Supplementary Figure 8: Comparison of mtDNAs of R498 and Nip. (**a**) Schematic diagram displaying sequence comparison and internal repeats of R498 and Nip mtDNAs. All sequences in Nip (490.5 kb) are aligned to R498 (527.1 kb). The Nip region 1-467,565 bp is aligned to R498 region 9,353-478,019 bp with sequence identity >99.5%. The last 23 kb in Nip was aligned to other regions of the Nip mtDNA. Alignment blocks (blue) within R498 or Nip show their large internal duplications (sequence identity >99%). There are two pairs of large repeats in the same direction and a set of three smaller repeats in R498, two of which are inverted repeats around 0 kb (note that the DNA is actually circular). (**b**) and (**c**) Screen shots taken from IGV show alignment of Nip PacBio reads (http://schatzlab.cshl.edu/data/ectools/) to Nip mtDNA, indicating that the two regions were misassembled. (**b**) The region around 51,835 bp corresponds to a 1 kb deletion compared to R498. (**c**) The region around 467,565 bp supports that the right end 23 kb of Nip is possibly a misplaced duplication.

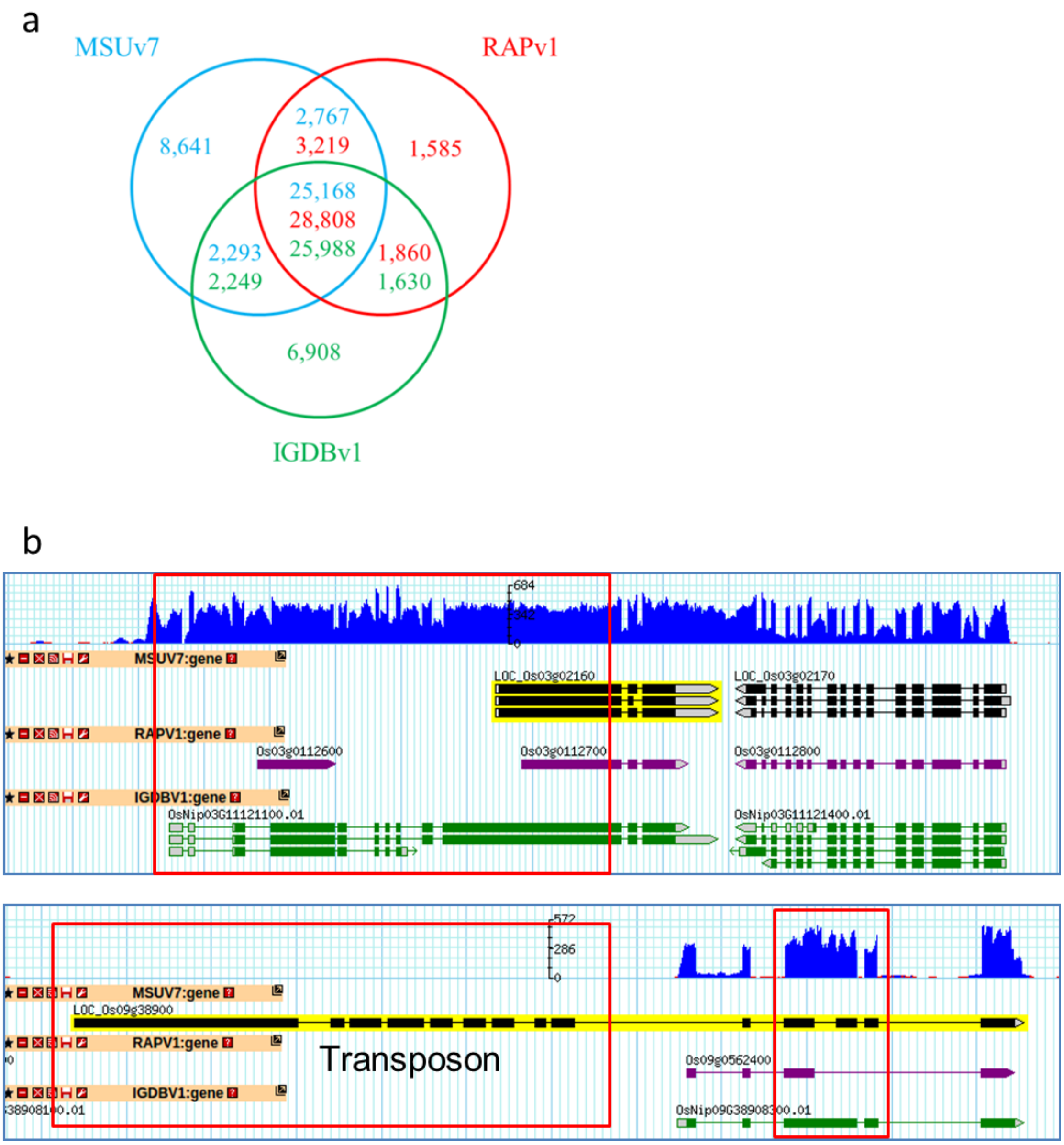

Supplementary Figure 9: Comparison of our annotated genes in Nip to existing reference gene sets. (**a**) Our Nip gene set (IGDBv1) contains 6,908 genes not overlapping with either MSU (MSUv7) or RAP (RAPv1) genes, while MSU and RAP contain 8,641 and 1,585 genes not overlapping with the other two sets, respectively. Among the overlapping genes, there are 24,616, 24,370, and 24,758 genes in MSU, RAP, and IGDB sharing the same exon-intron structure (with possibly different 5'-UTR start or 3'-UTR end) to the genes in at least one of the other two sets, respectively, suggesting their high similarity for the majority of the genes. The different number of overlapping genes for each set indicate a non 1:1 relationship, i.e., a gene in one set can overlap more than one gene in another set. (**b**) Examples of different overlapping genes annotated in the three gene sets. Both genes were incorrectly annotated in MSU and RAP based on RNA-seq evidence shown in blue.

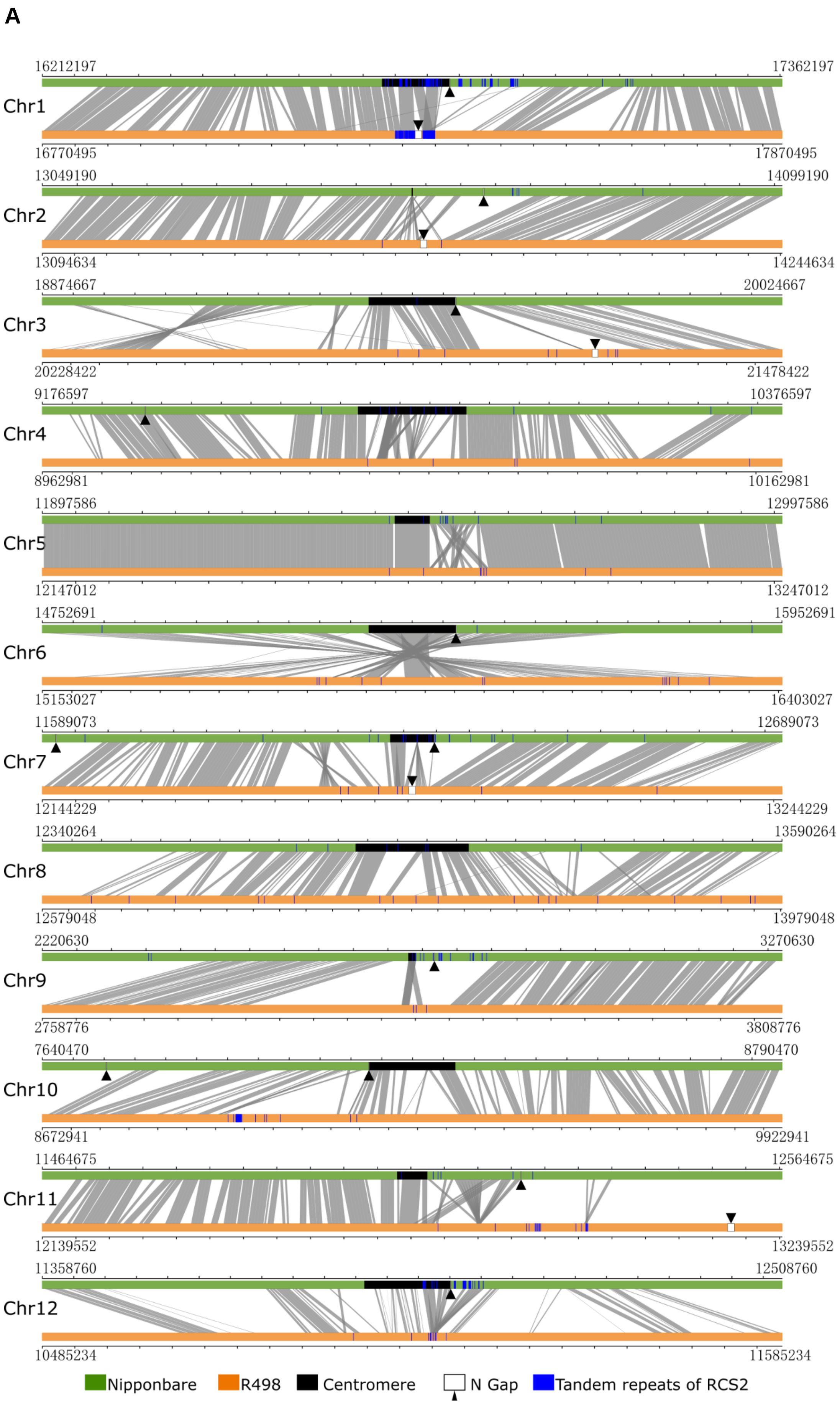

A
16212197
17362197
Chr1
16770495
17870495
13049190
14099190
Chr2
13094634
14244634
18874667
20024667
Chr3
20228422
21478422
9176597
10376597
Chr4
8962981
10162981
11897586
12997586
Chr5
12147012
13247012
14752691
15952691
Chr6
15153027
16403027
11589073
12689073
Chr7
12144229
13244229
12340264
13590264
Chr8
12579048
13979048
2220630
3270630
Chr9
2758776
3808776
7640470
8790470
Chr10
8672941
9922941
11464675
12564675
Chr11
12139552
13239552
11358760
12508760
Chr12
10485234
11585234
Nipponbare
R498
Centromere
N Gap
Tandem repeats of RCS2

B

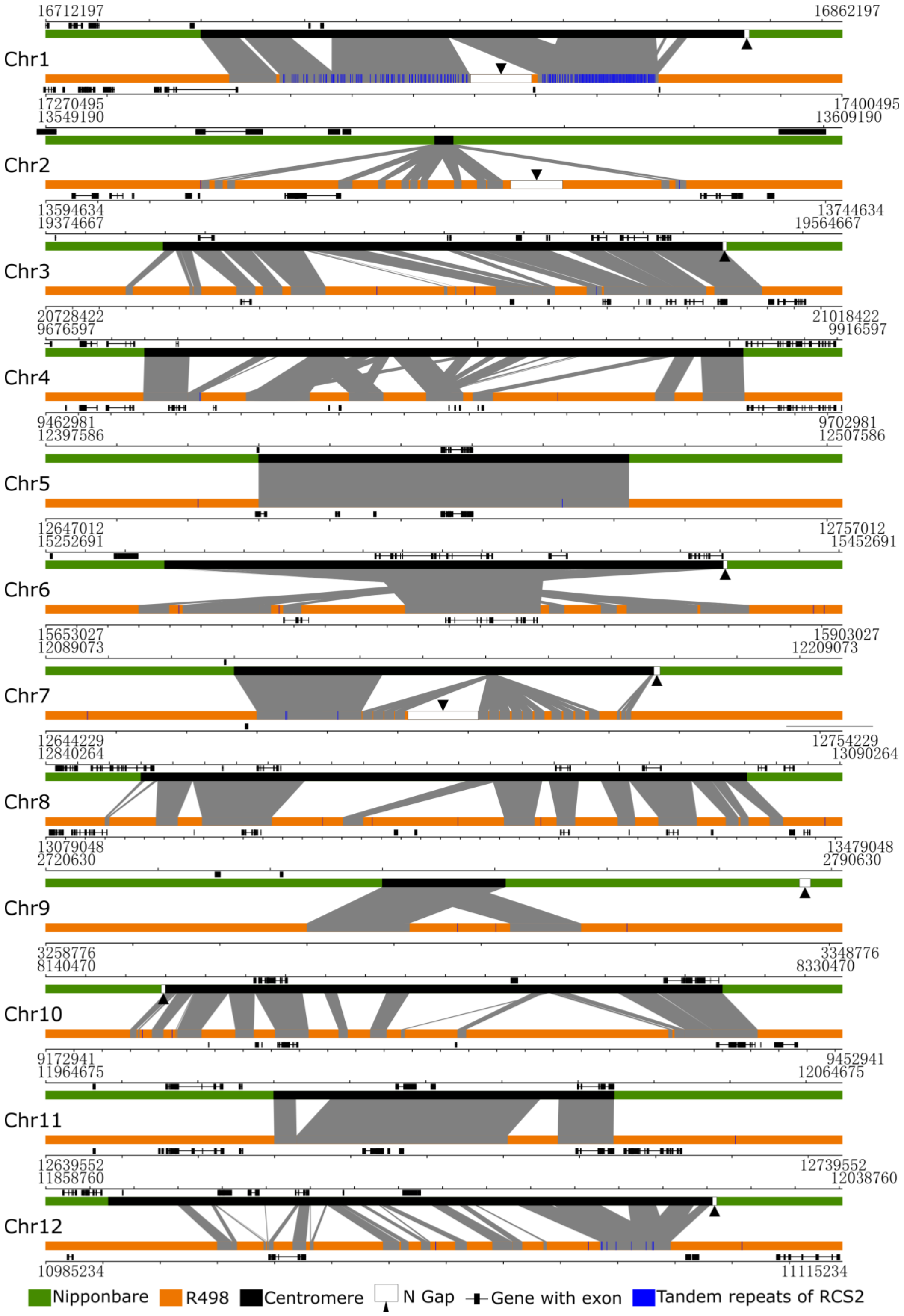

Supplementary Figure 10: Schematic displaying syntenic blocks of R498 to Nip in centromere regions. (**A**) All syntenic blocks in the regions were shown. (**B**) Only the alignments of Nip centromere-surrounding sequences (http://rice.plantbiology.msu.edu/annotation_pseudo_centromeres.shtml) to R498 sequences were displayed with annotated genes.

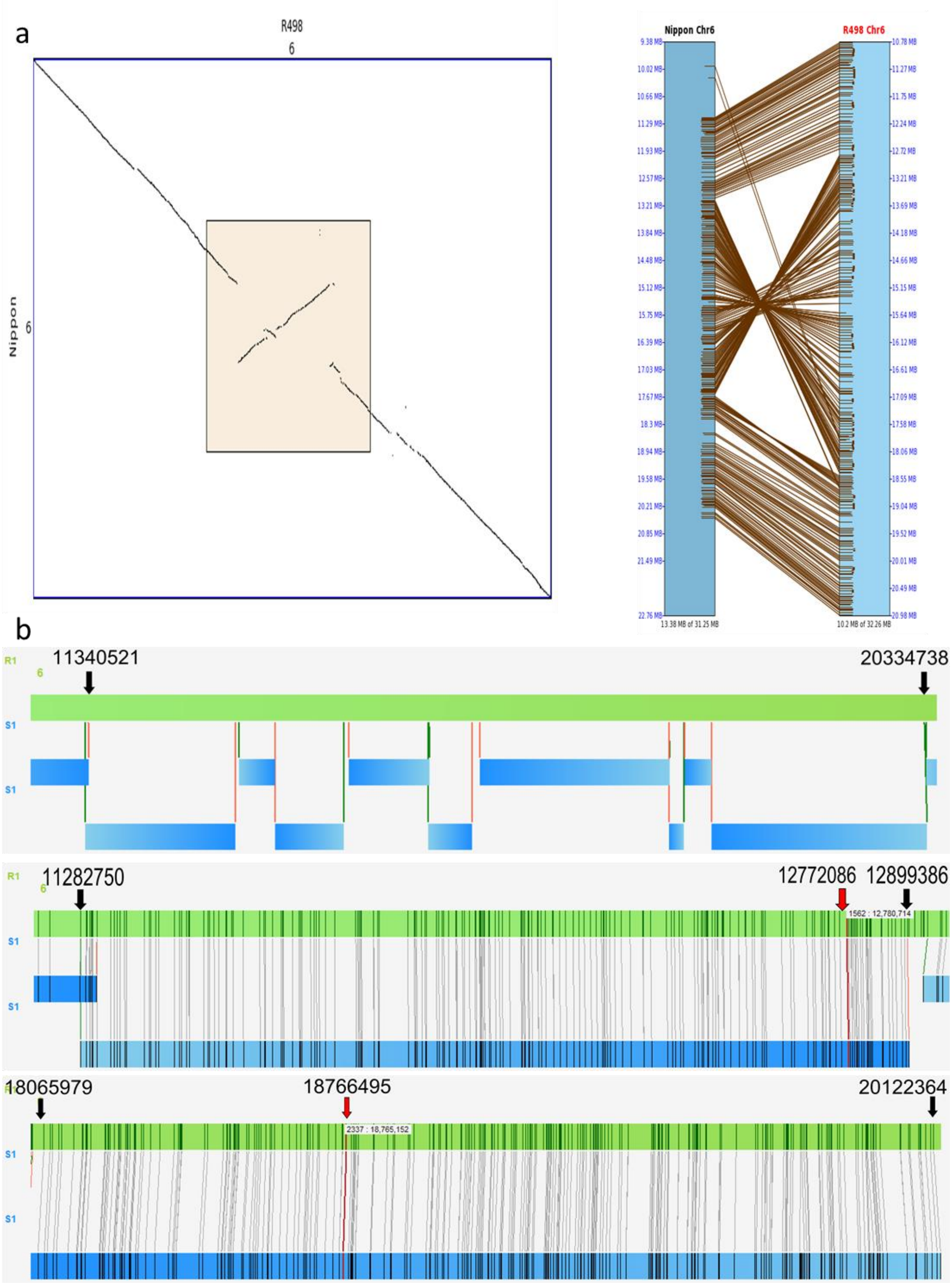
a
R498
6
Nippon
6
Nippon Chr6
R498 Chr6
13.38 MB of 31.25 MB
10.2 MB of 32.26 MB
b
11340521
20334738
11282750
12772086
12899386
1562 : 12,780,714
18065979
18766495
20122364
2337 : 18,765,152

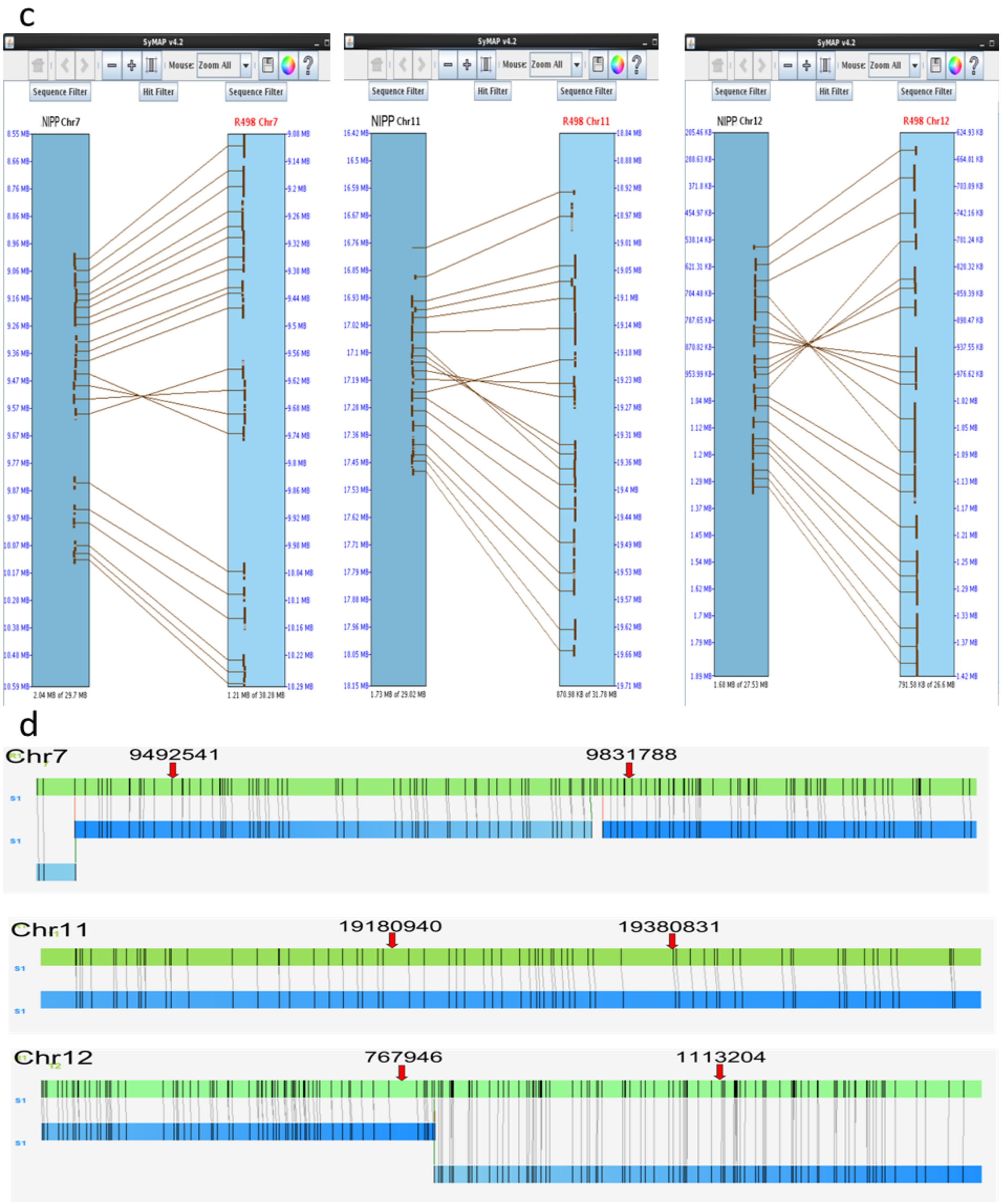

Supplementary Figure 11: Inversions between R498 and Nip. (**a**) Dot-matrix and synteny plot showing the inversion around the centromere of chromosome 6 from 12.76 Mb to 18.55 Mb between R498 and Nip. (**b**) Comparison of genome maps to R498 on chromosome 6 shows the correctness of the sequence assembly in R498. The first break point of the inversion (between 12.76-12.81 Mb) in R498 is supported by a single genome map. The second break point of inversion (between 18.51-18.55 Mb) in R498 supported by another single genome map. (**c**) Three smaller inversions on chromosomes 7, 11 and 12. (**d**) Comparison of the R498 sequences of the three inversions in (**c**) to genome maps shows that their boundaries (red arrows) in R498 were assembled correctly.

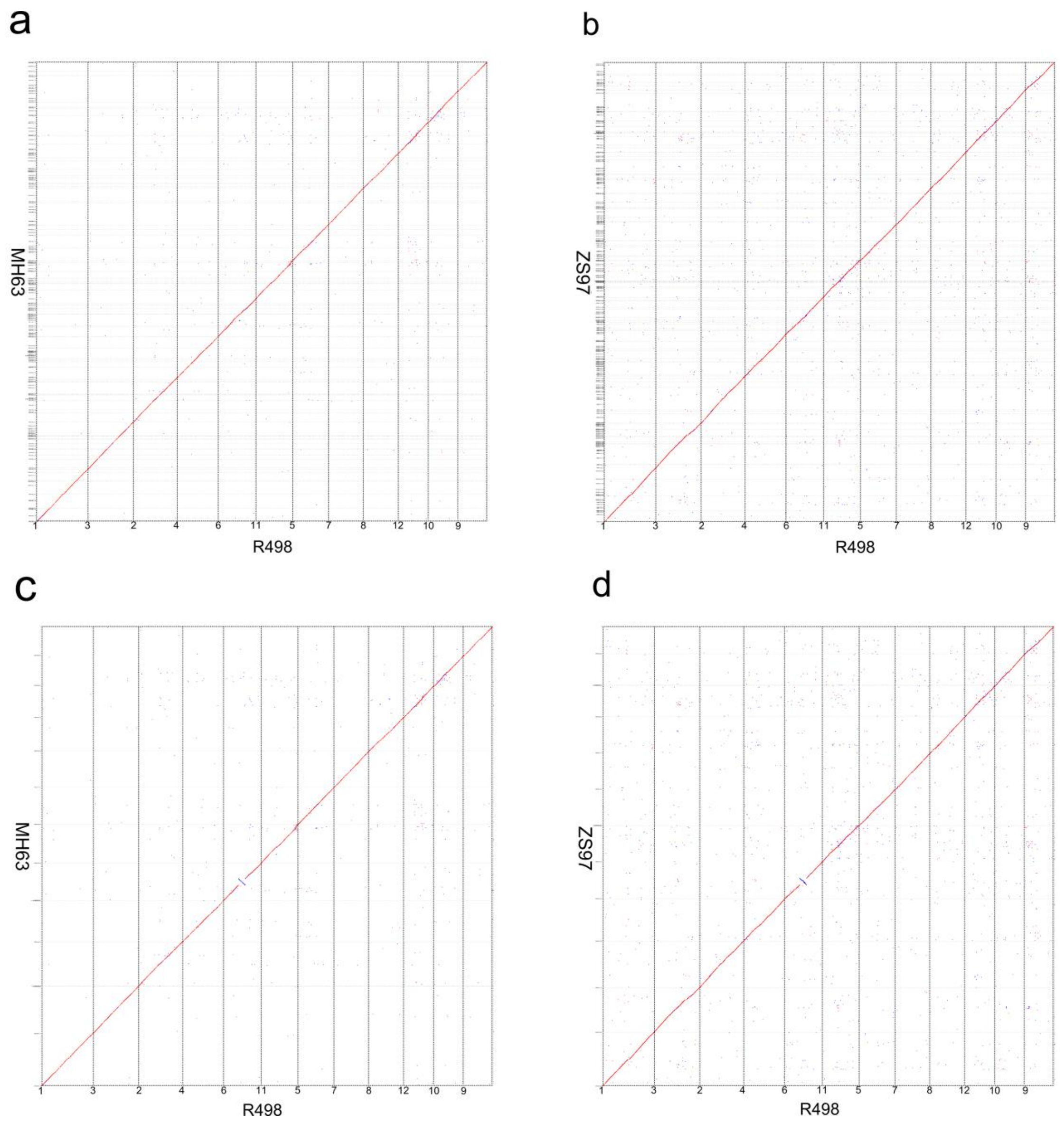

Supplementary Figure 12: Dot plots showing alignment of the MH63 and ZS97 sequences to R498. The x-axis represents R498 genome (0-390.3 Mb), and the y-axis represents MH63 in (a) or (c) (0-359.9 Mb) and ZS97 in (b) or (d) (0-346.8 Mb). (**a**) (**b**) Alignment of contig sequences to R498 genome. (**c**) (**d**) Alignment of chromosome sequences (pseudomolecules) to R498 genome. The numbers on x-axis are the chromosome numbers which are in the same order for y-axis. Both genomes of MH63 and ZS97 are highly syntenic to R498 at both contig level and chromosome level. The blue dotted lines in (c) and (d) represent the same inversion on chromosome 6 between R498 and Nip.

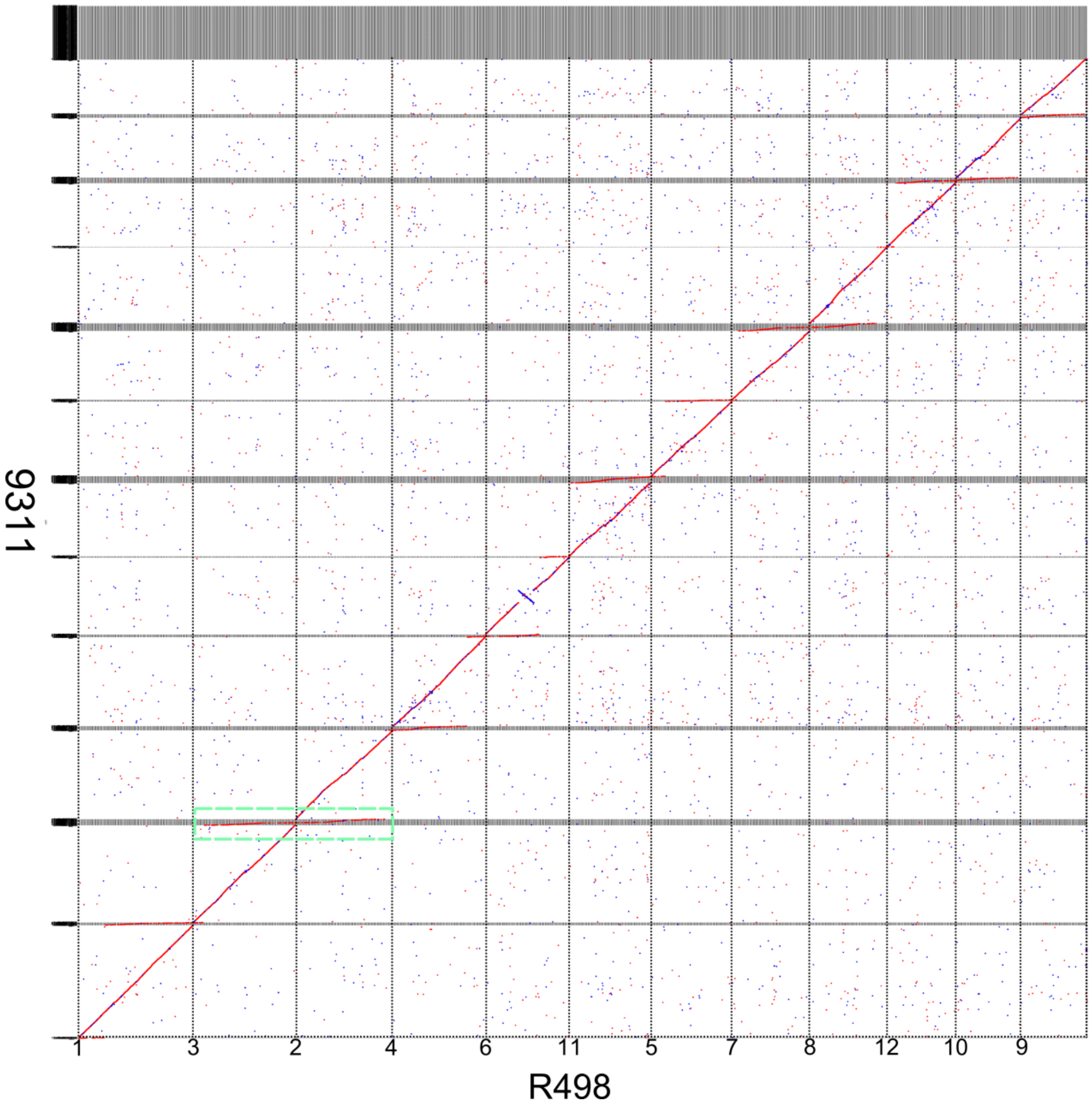

Supplementary Figure 13: Dot plot showing alignment of the 93-11 sequences to R498. The x-axis is the R498 sequences (0-390.3 Mb). The numbers on x-axis are the chromosome numbers which are in the same order for y-axis. Note that there are many 93-11 unanchored sequences being aligned to each chromosome of R498, represented by red dotted lines as the one bounded by the light blue dashed box. The blue dotted line represents the same inversion on chromosome 6 between R498 and Nip.

Supplementary Tables

Supplementary Table 1: Summary of sequencing data for R498 samples.

**Data source**	**Data size (Gb)**	**Read N50**	**Sample**
Genomic PacBio	47	11.2 kb	61 Cells
Fosmid	6.3	2x125 bp	576 Pools
Population	26.9	2x125 bp	364 F_3
BioNano	99	202 kb	5 Cells
Genomic Illumina	38.7	2x150 bp	1 Lane (SR)
RNA-seq	7.8	2x125 bp	Young hull 1 (H1)
RNA-seq	7.7	2x125 bp	Young hull 2 (H2)
RNA-seq	7.5	2x125 bp	Young leaf 1 (L1)
RNA-seq	8.5	2x125 bp	Young leaf 2 (L2)
RNA-seq	8	2x125 bp	Young spikelet 1 (SP1)
RNA-seq	7.9	2x125 bp	Young spikelet 2 (SP2)
RNA-seq	8.3	2x125 bp	Young stem (ST)
RNA-seq	8.7	2x125 bp	Root (R)

Supplementary Table 2: Statistics of the assembled contigs.

Assembly	Contig type	Size (Mb)	Contig #	N50 (bp)	Min (bp)	Max (bp)
PBcR HS	All	471.1	3,822	443,801	13,499	3,013,488
	Genome	450.6	3,266	470,500	13,499	3,013,488
	mtDNA+cpDNA	10.23	326	32,892	14,882	79,505
	Microbe	6.33	92	96,124	16,109	548,152
	Centromere	2.96	98	30,980	13,558	85,726
	Unclassified	1.03	40	26,405	17,708	44,939
CANU	All	405.1	1,226	899,904	4,129	3,454,773
	Genome	400.4	1,112	903,464	6,385	3,454,773
	mtDNA+cpDNA	1.77	29	66,557	8,533	122,339
	Microbe	2.95	84	39,576	4,129	93,147
	Centromere	0	-	-	-	-
	Unclassified	0.05	1	51,828	51,828	51,828
Falcon	All	397.2	3,460	516,065	1,003	3,830,954
	Genome	385.9	2,963	525,893	1,003	3,830,954
	mtDNA+cpDNA	5.42	464	14,318	1,088	61,985
	Microbe	5.66	19	1,689,908	1,076	2,164,409
	Centromere	0	-	-	-	-
	Unclassified	0.18	14	17,906	1,964	27,674
PBcR LS	All	436	2,045	1,145,810	12,178	10,316,720
	Genome	423.2	1,687	1,185,206	13,178	10,316,720
	mtDNA+cpDNA	5.97	182	34,553	14,990	96,724
	Microbe	4.37	76	68,575	15,646	233,386
	Centromere	1.58	64	24,751	13,838	74,592
	Unclassified	0.93	36	28,414	15,662	46,996
Fosmid contigs		39,700	1,215,918	42,048	10,000	616,771
SR-SOAPdenovo		477	1,954,935	2,527	64	120,702

Contig types: PBcR LS, PBcR assembly under low stringent conditions; PBcR HS, PBcR assembly under high stringent criteria; CANU, CANU assembly; Falcon, Falcon assembly; Fosmid contigs, assembled fosmid contigs from pooled fosmid clones; Centromere, indicating contigs containing centromeric satellites but not mapped onto the pseudomolecules; Unclassified, indicating the remaining contigs not mapped onto the pseudomolecules.

Supplementary Table 3: The uncorrected indels of >10 kb in the final pseudomolecules of R498 comparing with genome maps.

Chr	Start	End	Length	Indel type
3*	7,986,535	7,987,001	28,618	deletion
3	39,425,500	39,439,832	117,227	deletion
3	39,450,696	39,613,108	12,021	deletion
5	4,627,892	4,650,728	14,963	insertion
5	4,672,952	4,689,226	21,759	deletion
5	11,822,074	11,830,483	32,141	deletion
5	16,873,416	16,878,464	64,420	deletion
7	16,466,293	16,541,344	71,006	deletion
8	6,022,125	6,023,806	27,980	deletion
8*	6,195,389	6,248,808	10,780	deletion
8*	17,908,992	17,913,266	35,508	deletion
9	14,965,388	14,976,911	153,679	deletion
9	15,007,454	15,032,827	175,273	deletion
9	21,496,040	21,545,824	164,706	deletion

Chr, chromosome. The chromosome start and end define the boundaries between which each indel is found by comparing to genome maps. Insertions and deletions are all on pseudomolecule. *Potential indels that are present in the original PBcR HS contigs.

Supplementary Table 4: R498 genome base accuracy estimated with Illumina short reads.

Sample	Coverage	SNP			Indel			Error rate	Unaligned
		Homo	Heter	All	Homo	Heter	All		
H_1	19.81%	718	1,525	2,243	451	280	731	1.51E-5	3.87%
H_2	22.41%	677	1,812	2,489	519	278	797	1.37E-5	4.49%
L_1	20.39%	891	1,626	2,517	475	234	709	1.72E-5	4.26%
L_2	20.76%	876	1,645	2,521	510	229	739	1.71E-5	4.20%
SP_1	23.15%	836	2,002	2,838	539	270	809	1.52E-5	7.64%
SP_2	23.22%	740	1,925	2,665	558	258	816	1.43E-5	5.50%
ST	19.71%	687	1,554	2,241	445	204	649	1.47E-5	3.88%
R	20.96%	822	1,520	2,342	472	190	662	1.58E-5	13.51%
SR	99.88%	1,197	14,096	15,293	3,945	929	4,874	1.32E-5	5.20%

See Supplementary Table 1 for sample names. Coverage, percentage of genome covered by aligned short reads. Homo, homozygous; Heter, Heterozygous. Unaligned, percentage of short reads not aligned to genome. Only homozygous SNPs/Indels were used for base error calculation. Error rate is computed based on the covered portion of the genome (genome size: 390.3 Mb). Unaligned, the percentage of short reads not aligned to the genome. Please note that many of the SNPs/Indels are possibly errors in short reads introduced by PCR or genetic variations between R498 samples. Therefore, the error rate is only an upper bound estimate.

Supplementary Table 5: Statistics of sequence sensitivity, specificity, redundancy and error rate of the WGS assemblies.

Assembly	CGL (Mb)	Sn (%)*	ACL (Mb)	Redu (%)	OCN (#)	COL (Mb)	Err (%)
PBcR HS	390.0	99.75	451.8	15.86	578	2.6	0.57
Falcon	368.4	94.22	387.9	5.29	415	5.6	1.38
CANU	389.7	99.68	400.2	2.68	213	4.3	1.07
PBcR LS	390.4	99.84	422.8	8.3	782	13.9	3.28
SR-SOAPdenovo	390.7	99.94	466.2	19.45	13	0.014	0.003

Sn, Sensitivity = CGL/genome length; Redu, Redundancy = (ACL-CGL)/CGL; Err, Error = COL/ACL. *Genome size: 390,983,850 bp.

CGL: Covered genome length by WGS contigs; ACL: total aligned contig length of a WGS assembly; OCN: overhang contig number, the number of contigs aligned to pseudomolecules with overhang length >1 kb; COL: sum of contig overhang length. If a contig is aligned to genome in multiple regions, it is designated as a chimeric contig, in which the aligned fragments except the longest one are treated as overhangs of the contig.

Supplementary Table 6: Statistics of the predicted protein-coding genes in R498 and Nip

Type	Nip			R498
	IGDBv1	MSUv7	RAPv1	IGDBv1
Gene number	36,775	38,869	35,472	38,714
Gene length*	2,408/3,191	2,188/2,855	2,458/3,082	2,292/3,012
Transcript length*	1,330/1,513	1,246/1,422	1,392/1,557	1,296/1,480
Max cds length	14,900	16,310	16,030	14,214
CDS length*	813/1,030	849/1,062	801/991	765/996
Protein length*	270/343	283/354	267/330	254/331
Exon length*	173/340	165/328	177/358	183/342
Intron length*	153/438	174/420	155/438	152/419
5' UTR length*	112/211	133/213	107/252	116/211
3' UTR length*	271/336	335/416	279/441	274/340
Exon number*	3.0/4.5	3.0/4.3	3.0/4.4	2.0/4.3
Intron number*	2.0/3.5	2.0/3.3	2.0/3.4	1.0/3.3

* Each number indicates the median/average.

Supplementary Table 7: Statistics of the functionally annotated genes in R498 and Nip

	Nip		R498	
	Number	%	Number	%
InterPro	27,846	75.72	27,269	70.44
GO	19,822	53.9	19,506	50.38
Pathway	4,259	11.58	4,133	10.68
Functionally annotated	32,385	88.06	32,839	84.82
Other	4,390	11.94	5,875	15.18
Total	36,775	100	38,714	100

Supplementary Table 8: Statistics of repeat content in R498 and Nip

	Nip		R498	
	Length (bp)	Percentage of genome (%)	Length (bp)	Percentage of genome (%)
Class I: Retrotransposon	105,098,791	28.16	117,509,061	30.05
LTR-Retrotransposon	98,903,987	26.5	111,173,484	28.43
LTR/Gypsy	65,915,787	17.66	80,330,011	20.55
LTR/Copia	17,931,866	4.8	15,079,454	3.86
LTR/Other	15,056,334	4.03	15,764,019	4.03
Non-LTR Retrotransposon	6,194,804	1.66	6,335,577	1.62
SINE	796,311	0.21	848,061	0.22
LINE	5,398,493	1.45	5,487,516	1.4
Class II: DNA Transposon	40,716,340	10.91	40,743,536	10.42
EnSpm/CACTA	14,117,095	3.78	13,264,041	3.39
hAT	1,641,580	0.44	1,897,505	0.49
Harbinger	3,729,352	1	3,882,866	0.99
Tc1/Mariner	462,697	0.12	607,903	0.16
MuDR	5,872,349	1.57	5,993,554	1.53
Helitron	1,850,232	0.5	1,702,664	0.44
Other	13,043,035	3.49	13,395,003	3.43
Other tandem repeat	3,935,022	1.05	4,548,484	1.16
Low Complexity	22,478	0.01	17,672	0
Unclassified	1,117,819	0.3	1,607,036	0.41
Total content	150,890,450	40.43	164,425,789	42.05

Supplementary Table 9: The number of genes in the centromere regions and subtelomere regions in R498 and their corresponding regions in Nip.

R498				Nipp			
Chr	Start	End	Gene	Chr	Start	End	Gene
Chr1	17,309,317	17,370,574	1	Chr1	16,745,001	16,820,731	2
Chr2	13,624,446	13,717,125	1	Chr2	13,587,767	13,755,012	1
Chr3	20,758,422	21,241,752	19	Chr3	19,405,002	19,580,034	9
Chr4	9,510,378	10,398,695	48	Chr4	9,744,101	10,600,528	37
Chr5	12,668,460	13,002,159	18	Chr5	12,416,072	12,739,602	14
Chr6	15,619,708	16,540,430	24	Chr6	14,653,526	15,487,462	29
Chr7	12,592,770	13,069,332	20	Chr7	12,032,145	12,698,352	22
Chr8	12,564,579	13,931,382	46	Chr8	12,542,754	13,640,031	44
Chr9	3,306,072	3,326,144	0	Chr9	2,826,031	2,847,921	0
Chr10	8,995,124	9,218,353	4	Chr10	8,120,001	8,184,428	0
Chr11	12,730,027	12,954,276	1	Chr11	12,068,946	12,280,001	7
Chr12	10,964,260	11,107,516	12	Chr12	11,766,940	11,954,396	17
Chr1	14,714	200,000	34	Chr1	1,133	183,501	30
Chr2	8,568	200,000	35	Chr2	1	200,001	35
Chr3	7,785	200,000	28	Chr3	1,001	195,286	29
Chr4	39,363	200,000	9	Chr4	5,899	175,489	6
Chr5	28,491	200,000	19	Chr5	1	173,672	18
Chr6	13,466	200,000	29	Chr6	118,309	309,293	26
Chr7	104,956	200,000	13	Chr7	1,132	95,386	12
Chr8	31,853	200,000	22	Chr8	8,024	170,386	24
Chr9	135,343	200,000	2	Chr9	146,901	257,717	2
Chr10	142,768	200,000	0	Chr10	45,293	60,341	2
Chr11	34,961	200,000	22	Chr11	47,284	215,024	21
Chr12	74,647	200,000	12	Chr12	2,280	108,733	14
Chr1	44,161,539	44,361,539	29	Chr1	43,083,653	43,255,001	25
Chr2	37,564,328	37,764,328	24	Chr2	35,785,001	35,936,248	21
Chr3	39,491,490	39,691,490	0	Chr3	0	0	0
Chr4	35,649,732	35,849,732	31	Chr4	35,400,861	35,502,695	18
Chr5	31,037,231	31,237,231	19	Chr5	29,650,362	29,840,001	21
Chr6	32,265,040	32,465,040	24	Chr6	31,050,764	31,219,275	23
Chr7	30,077,827	30,277,827	19	Chr7	29,510,237	29,672,673	19
Chr8	29,752,003	29,952,003	9	Chr8	28,370,262	28,423,759	9
Chr9	24,560,661	24,760,661	25	Chr9	22,790,249	22,940,001	20
Chr10	25,382,588	25,582,588	10	Chr10	0	0	0
Chr11	31,578,392	31,778,392	0	Chr11	29,000,021	29,009,437	1
Chr12	26,401,357	26,601,357	22	Chr12	27,395,028	27,530,857	24

Centromere regions containing all the full units of rice centromere tandem repeats RCS2.

Supplementary Table 10: rDNA (17S-5.8S-25S) repeats in R498 and Nip.

Genome	Chr	Start	End	Length	rDNA Type	Identity
R498	Chr10	10569	12216	1648	17s	99.21%
R498	Chr10	12305	12588	284	5.8S	91.52%
R498	Chr10	12803	16187	3385	25S	98.76%
R498	Chr10	18989	20800	1812	17s	99.34%
R498	Chr10	20889	21172	284	5.8S	91.52%
R498	Chr10	21384	24768	3385	25S	98.76%
R498	Chr10	27841	29651	1811	17s	99.28%
R498	Chr10	29740	30019	280	5.8S	91.79%
R498	Chr10	30230	33592	3363	25S	98.03%
R498	Chr10	36146	37951	1806	17s	99.01%
R498	Chr10	38040	38322	283	5.8S	91.17%
R498	Chr10	38537	41908	3372	25S	98.50%
R498	Chr10	44464	46266	1803	17s	98.84%
R498	Chr10	46355	46636	282	5.8S	91.49%
R498	Chr10	46844	50215	3372	25S	98.38%
R498	Chr10	52778	54582	1805	17s	98.95%
R498	Chr10	54720	54941	222	5.8S	98.65%
R498	Chr10	55154	58528	3375	25S	98.29%
R498	Chr10	61051	62845	1795	17s	98.78%
R498	Chr10	62934	63217	284	5.8S	91.52%
R498	Chr10	63429	66783	3355	25S	97.99%
R498	Chr10	69388	71195	1808	17s	99.12%
R498	Chr10	71283	71566	284	5.8S	91.52%
R498	Chr10	71777	75149	3373	25S	98.26%
R498	Chr10	77748	79548	1801	17s	98.13%
R498	Chr10	79637	79918	282	5.8S	91.49%
R498	Chr10	80128	83458	3331	25S	96.64%
R498	Chr10	85971	87772	1802	17s	98.73%
R498	Chr10	87864	88138	275	5.8S	90.25%
R498	Chr10	88349	91687	3339	25S	96.36%
R498	Chr10	94189	95994	1806	17s	99.01%
R498	Chr10	96082	96364	283	5.8S	91.17%
R498	Chr10	96578	99904	3327	25S	97.02%
R498	Chr10	102486	104289	1804	17s	98.79%
R498	Chr10	104377	104656	280	5.8S	91.46%
R498	Chr10	104868	108239	3372	25S	98.20%
R498	Chr10	110325	112123	1799	17s	98.46%
R498	Chr10	112206	112490	285	5.8S	91.20%
R498	Chr10	112694	116047	3354	25S	97.50%
R498	Chr10	118825	120636	1812	17s	99.34%
R498	Chr10	120725	121008	284	5.8S	91.52%

R498	Chr11	26262484	26262652	169	17s	98.21%
R498	Chr5	3969104	3969694	591	25S	91.53%
R498	Chr6	8437060	8437402	343	25S	91.52%
R498	Chr7	12101977	12103274	1298	25S	95.78%
R498	Chr7	12103289	12104465	1177	25S	96.26%
R498	Chr9	10566	12211	1646	17s	98.85%
R498	Chr9	12298	12581	284	5.8S	91.52%
R498	Chr9	12795	16177	3383	25S	98.64%
R498	Chr9	19137	20941	1805	17s	98.95%
R498	Chr9	21030	21313	284	5.8S	91.52%
R498	Chr9	21525	24883	3359	25S	97.88%
R498	Chr9	27127	28938	1812	17s	99.34%
R498	Chr9	29027	29310	284	5.8S	91.52%
R498	Chr9	29525	32910	3386	25S	98.79%
R498	Chr9	35508	37318	1811	17s	99.06%
R498	Chr9	37406	37689	284	5.8S	91.52%
R498	Chr9	37901	41266	3366	25S	98.20%
R498	Chr9	43797	45607	1811	17s	98.84%
R498	Chr9	45695	45972	278	5.8S	91.34%
R498	Chr9	46182	49528	3347	25S	97.61%
R498	Chr9	52086	53897	1812	17s	99.34%
R498	Chr9	53985	54268	284	5.8S	91.52%
R498	Chr9	54483	57868	3386	25S	98.79%
Nip	Chr9	1000	1480	480	25S	98.13%
Nip	Chr9	3625	5436	1811	17s	99.45%
Nip	Chr9	6023	9408	3385	25S	98.79%
Nip	Chr9	11553	13364	1811	17s	99.45%
Nip	Chr9	13951	17336	3385	25S	98.79%
Nip	Chr9	19481	21292	1811	17s	99.45%
Nip	Chr9	21879	25264	3385	25S	98.79%
Nip	Chr9	27409	29220	1811	17s	99.45%
Nip	Chr9	29807	33192	3385	25S	98.79%
Nip	Chr9	35337	37148	1811	17s	99.45%
Nip	Chr5	3893143	3893733	590	25S	91.02%
Nip	Chr6	8367093	8367435	342	25S	92.11%
Nip	Chr11	23702822	23702990	168	17s	97.62%
Nip	Chr2	28715349	28716992	1643	25S	98.24%
Nip	Chr2	28719356	28721162	1806	17s	98.89%
Nip	Chr2	28721256	28721537	281	5.8S	92.53%
Nip	Chr2	28721753	28721984	231	25S	99.57%

Supplementary Table 11: Alignment between R498 and Nip around the boundaries of inversion on chromosome 6.

Chr	R498_start	R498_end	Nip_start	Nip_end	R498_length	Nip_length	Identity
6	12,743,460	12,758,973	13,051,949	13,067,468	15,514	15,520	98
**6**	**12,760,622**	**12,762,376**	**13,069,631**	**13,071,448**	**1,755**	**1,818**	**95**
**6**	**12,817,462**	**12,819,558**	**17,632,495**	**17,630,424**	**2,097**	**2,072**	**95**
6	12,824,000	12,832,609	17,622,992	17,614,390	8,610	8,603	98
6	12,832,462	12,841,815	17,614,391	17,605,012	9,354	9,380	99
6	12,841,733	12,853,219	17,604,768	17,593,263	11,487	11,506	99
6	12,853,696	12,858,703	17,590,727	17,585,709	5,008	5,019	99
6	12,859,679	12,872,169	17,585,700	17,573,120	12,491	12,581	97
6	18,435,021	18,500,300	13,200,518	13,135,281	65,280	65,238	98
6	18,501,312	18,506,480	13,133,431	13,128,253	5,169	5,179	97
6	18,506,672	18,511,650	13,127,528	13,122,409	4,979	5,120	95
**6**	**18,512,446**	**18,514,113**	**13,121,348**	**13,119,682**	**1,668**	**1,667**	**99**
**6**	**18,551,092**	**18,615,997**	**17,853,290**	**17,788,273**	**64,906**	**65,018**	**98**
6	18,615,992	18,620,537	17,787,926	17,783,337	4,546	4,590	97
6	18,620,936	18,649,662	17,782,802	17,753,990	28,727	28,813	98

The first boundary on R498 is between 12.76 Mb and 12.82 Mb; the second boundary on R498 is between 18.51 Mb and 18.56 Mb. Chr, chromosome.

Supplementary Table 12: Go enrichment analysis of genes enclosed in the PVs between R498 and Nip.

**GO enrichment of genes in R498 PVs relative to Nip**					
**ID**	**P-value**	**Q-value**	**Go category**	**Go term**	**Significance**
GO:0015074	3.39E-16	2.98E-14	biological process	DNA integration	***
GO:0006468	5.14E-11	1.29E-09	biological process	protein phosphorylation	***
GO:0000723	1.32E-07	2.91E-06	biological process	telomere maintenance	***
GO:0006383	1.19E-04	1.79E-03	biological process	transcription from RNA polymerase Ⅲ promoter	**
GO:0006281	1.07E-03	1.25E-02	biological process	DNA repair	*
GO:0005666	0.00011938	0.001786566	cellular component	DNA-directed RNA polymerase Ⅲ complex	**
GO:0043531	2.64E-21	4.65E-19	molecular function	ADP binding	***
GO:0003676	7.90E-15	4.64E-13	molecular function	nucleic acid binding	***
GO:0016772	2.28E-12	1.00E-10	molecular function	transferase activity, transferring phosphorus-containing groups	***
GO:0004672	4.81E-11	1.29E-09	molecular function	protein kinase activity	***
GO:0004674	4.91E-11	1.29E-09	molecular function	protein serine/threonine kinase activity	***
GO:0003678	3.79E-07	7.41E-06	molecular function	DNA helicase activity	***
GO:0005524	0.000121811	0.001786566	molecular function	ATP binding	**
GO:0030247	0.000170825	0.002312706	molecular function	polysaccharide binding	**
GO:0004523	0.000224372	0.002820672	molecular function	RNA-DNA hybrid ribonuclease activity	**
**GO enrichment of genes in Nip PVs relative to R498**					
**ID**	**P-value**	**Q-value**	**Go category**	**Go term**	**Significance**
GO:0015074	3.98E-22	2.90E-20	biological process	DNA integration	***
GO:0019684	3.69E-05	0.000768802	biological process	photosynthesis, light reaction	***
GO:0009772	0.00014414	0.002630558	biological process	photosynthetic electron transport in photosystem Ⅱ	**

GO:0035556	0.001781665	0.023647556	biological process	intracellular signal transduction	*
GO:0003676	2.66E-34	3.89E-32	molecular function	nucleic acid binding	***
GO:0004523	3.50E-11	1.70E-09	molecular function	RNA-DNA hybrid ribonuclease activity	***
GO:0043531	2.78E-06	0.000100078	molecular function	ADP binding	***
GO:0008234	3.43E-06	0.000100078	molecular function	cysteine-type peptidase activity	***
GO:0016651	4.37E-06	0.000106253	molecular function	oxidoreductase activity, acting on NAD(P)H	***
GO:0045156	0.000189336	0.003071454	molecular function	electron transporter, transferring electrons within the cyclic electron transport pathway of photosynthesis activity	**
GO:0008137	0.000214183	0.003127069	molecular function	NADH dehydrogenase (ubiquinone) activity	**
GO:0047134	0.003717539	0.045230052	molecular function	protein-disulfide reductase activity	*
GO:0048038	0.004108067	0.046136755	molecular function	quinone binding	*

Supplementary Table 13: AGP file of the corrected regions >10 kb with added WGS contigs.

Indel	Start	End	Comp Num	Type	Comp Id	Comp Start	Comp End	Orient
1	1	976914	1	W	Ctg901	1	976914	+
1	986389	1010336	2	I	Ctg902	9474	33422	+
1	1016279	2203446	3	W	Ctg1046	4	1187171	+
2	1	349255	1	W	Ctg299	1	349255	−
2	357032	398646	2	I	Ctg146	132	41746	+
2	409328	633644	3	W	Ctg152	4	224320	+
3	1	251602	1	W	Ctg149	1	251602	+
3	259328	284243	2	I	Ctg725	8	24923	+
3	310321	704515	3	W	Ctg724	26078	420272	+
4	1	801230	1	W	Ctg1207	1	801230	+
4	813682	846044	2	I	Ctg345	1292	33654	−
4	873927	1414417	3	W	Ctg283	36219	576709	+
5	1	231474	1	W	Ctg8308	1	231474	+
5	242827	248830	2	I	Ctg8309	19032	25035	+
5	254591	275017	3	I	Ctg8606	3	20429	−
5	275090	629314	4	W	Ctg9353	78	354302	−
6	1	193072	1	W	Ctg1624	1	193072	+
6	197027	227775	2	I	Ctg1707	3	30751	−
6	227932	262848	3	I	Ctg336	1	34916	+
6	271249	424918	4	W	Ctg1628	16231	169900	+
7	1	381372	1	W	Ctg11	1	381372	+
7	392762	427426	2	I	Ctg7915	12	34676	+
7	427510	1459182	3	W	Ctg1061	1	1031672	+
8	1	260320	1	W	Ctg6736	1	260320	+
8	260986	280479	2	I	Ctg6737	1	19493	+
8	280932	299324	3	I	Ctg6738	3	18395	+
8	299832	340125	4	I	Ctg6739	128	40421	+
8	341901	371692	5	I	Ctg4737	2	30001	−
8	390218	612117	6	W	Ctg7508	3	221902	−
9	1	203132	1	W	Ctg8356	1	203132	+
9	207518	235718	2	I	Ctg8355	19	28219	+
9	239481	265599	3	I	Ctg8354	3	26121	+
9	269761	295683	4	I	Ctg8323	2	25924	−
9	297840	536323	5	W	Ctg4697	517	239000	−
10	1	1009634	1	W	Ctg816	1	1009634	+
10	1029397	1057514	2	I	Ctg247	16321	44438	−
10	1059328	1334937	3	W	Ctg817	7	275616	+
11	1	1548870	1	W	Ctg1053	1	1548870	+

11	1549312	1582788	2	I	Ctg1056	5	33481	+
11	1587820	1803115	3	W	Ctg1058	14	215309	+
12	1	296194	1	W	Ctg1519	1	296194	+
12	296834	339964	2	I	Ctg186	9	43130	−
12	340917	763245	3	W	Ctg851	7	422329	−
13	1	276682	1	W	Ctg8840	1	276682	+
13	277963	330845	2	I	Ctg6112	4	52886	+
13	339712	373031	3	I	Ctg8443	9	33328	−
13	376291	404595	4	I	Ctg8444	2	28306	−
13	406012	553221	5	W	Ctg8601	6219	153428	+
14	1	1092655	1	W	Ctg72	1	1092655	+
14	1099465	1156220	2	I	Ctg208	76	56831	−
14	1170328	1652206	3	W	Ctg113	8932	490810	+
15	1	288727	1	W	Ctg46	1	288727	+
15	289176	352398	2	I	Ctg7750	19	63241	−
15	354921	474481	3	W	Ctg105	42	119602	+

Indel, the regions containing an indel. Start and End, starting and ending positions for the contig sequences in the regions. The gaps between contigs were filled with fosmid contigs. Comp, contig component in each region. W, the boundary contigs for each region; I, newly added contigs in each region. Comp Start and End, defined the starting and end positions of the contig sequences used in the region. Orient, the orientation of the contigs in the final sequences.

Supplementary Notes

Supplementary Note 1: Why and how does the contig connection process work?

The process of building super-contigs can be viewed as to connect or extend WGS contigs on the genetic map iteratively with unanchored WGS contigs and fosmid contigs by merging the nodes on overlap graph (Fig. 1).

Each fosmid clone pool contains about 10% random portion of the genome, in which many segmentally duplicated regions become single-copy regions. These regions can be correctly assembled in random fosmid pools. It is possible to assemble chimeric contigs in each pool, but for each region, there should be always more correctly assembled contigs than incorrect ones. Thus, between any node pair, if they are truly immediately adjacent, there should be many fosmid contigs to connect them. For random node pairs, there should be only a small number of chimeric fosmid contigs to connect them. Therefore, the node pairs with many fosmid links with high quality overlaps should be connected first; we used a generalized score (weighed score; see Methods) to prioritize the node pairs to be merged.

All links between a pair of nodes on the overlap graph are created based on their overlaps to the opposite ends of fosmid contigs. However, a link can be viewed as one of the following subtypes based on the genetic map positions of the nodes it connects: (1) direct link (negative edge length) between a pair of neighboring WGS contigs with sequence overlap between the WGS contigs; (2) indirect link (positive edge length) between two neighboring WGS contigs without sequence overlap between the WGS contigs; (3) direct or indirect link between an anchored WGS contig and an unanchored WGS contig; (4) direct or indirect link between two unanchored WGS contigs. Clearly, the positional information on a genetic map has put another restriction which can be used to prioritize the node pairs to be merged, ie, the closer a node pair on a linkage group, the less chance of error can occur when connecting them. For example, a direct link is generally more reliable than an indirect link, and the links involving anchored contigs are generally more reliable than those not.

Compared with randomly connecting two nodes with a fosmid link between them, we can see that by utilizing the restrictions that fosmid contigs and genetic map put on the overlap graph, the success rate of connecting two truly adjacent WGS contigs will be increased significantly. We minimized the connection errors by observing the following rules: (1) non-redundancy rule, i.e. a WGS contig is used only once; the used contig (and all its links from the used end to the same linkage group and all links to different linkage groups) is removed to reduce conflicts; (2) global best-match-first rule, i.e., the best-scored link (in all linkage groups) is merged first; (3) delayed conflict-resolving rule, i.e., if a contig end overlaps with more than one WGS contigs with the same score, first anchored nodes, then nodes with direct links are merged first; otherwise the connection is delayed to next step; (4) decreasing and minimum overlap threshold rule, i.e., a sequence overlap cannot be shorter than 5 kb with a minimum alignment identity of 97% and three level of identity threshold (99%, 98% and 97%) are used in turn to merge the nodes satisfying each threshold. No nodes from the different linkage groups can be merged except the split part from chimeric contigs.

On the overlap graph constructed using PBcR HS contigs, we found that the average edge number between the nodes on the path of the final super-contigs was 29, while the average edge number for all other connected node pairs being merely 2. As a comparison, the average edge number between the nodes (contigs) adjacent on hybrid genome maps was 27, and the average edge number between other contigs on hybrid maps was also 2. Totally 81% of the gaps in hybrid maps can be connected by one fosmid contig. These results indicated that the fosmid contigs are mostly correctly assembled with a small number of random chimeric ones which did not affect the quality of the connecting regions.

Initially we used the PBcR LS contigs to build super-contigs since the genetic map was constructed using LS contigs. However, after several iterations when no more neighboring contigs (or super-contigs) could be connected, we found that the unfilled

gaps between adjacent contigs (or super-contigs) were either located in the centromere regions or resulted from misassembled LS contigs around the gaps. For the latter case, we found that those gaps could be successfully filled after replacing the PBcR LS sequences around the gaps with the best matched HS contigs. Therefore, we redid the whole connection process by aligning the HS contigs onto the constructed LS super-contigs to group and order them (to form anchored and unanchored HS contig sets) and using the fosmid contigs to connect them. In the final HS super-contigs, no unfilled gaps were left due to misassembled contigs. The replacement of PBcR LS contigs with HS contigs also added a few Mb of sequences into the final super-contigs which included several more telomeres. These results suggested that although the LS assembly had higher N50 size but it contained many errors not existing in the HS assembly which was more accurate for the final assembly.

Supplementary Note 2: Quality assessment of the WGS assemblies and the connecting regions

Based on the best aligned blocks of the assembled WGS contigs to the pseudomolecules, we computed the following metrics to evaluate the quality of the WGS assemblies: sensitivity (length percentage of genome being covered by WGS contigs), redundancy ((total aligned length of WGS contigs – covered genome length) / covered genome length) and error rate (total overhang length of >1 kb of aligned WGS contigs / total aligned length of WGS contigs) (Supplementary Table 5). The PBcR HS assembly had the highest sensitivity (99.75%), the highest redundancy (15.86%) and the lowest error rate (0.57%). The high sequence redundancy and low error rate in the PBcR assembly suggested that it generated multiple copies of sequences in repetitive (or heterozygous) regions (Supplementary Fig. 5), which represented a general feature of overlap-layout-consensus (OLC) assemblers[1], such as Celera in PBcR. On the other hand, string graph assemblers such as Falcon do not usually keep repetitive sequences at the end of assembled contigs[1]. These results suggested that the PBcR HS contigs were very suitable for building super-contigs under the guidance of a genetic map or genome map.

The PBcR HS contigs covered 98.34% of the connecting regions with identity >=98%, indicating that most of the connecting sequences were present in HS assembly, but in short contigs with N50 size of 24 kb. The Illumina short reads covered 98.02% of the connecting regions (with sequencing depth of 109x) with identity>=98%. As a comparison, the whole genome coverage and sequencing depth from Illumina short reads was 99.88% and 89x. The high sequencing depth in the connecting regions suggested that they were unlikely to be misassembled at base level. If misassembled, the low quality regions should have much lower sequencing depth or sequencing coverage (see Supplementary Fig. 7). Illumina platform cannot sequence many GC-biased regions. The total number of zero mapping regions by Illumina short reads were 4,751 (a total of 486,331 bp), with max length of 10,170 bp. Since these regions

are shorter than the read length of SMRT sequences, they were very unlikely caused by misassemblies.

Supplementary Note 3: Estimation of R498 genome size

The aligned raw SMRT reads to pseudomolecules was 98.54%, covering 99.99% of the genome. After PBcR self-correction, we obtained 16.2 Gb of corrected sequences, of which 99.94% were aligned to the final pseudomolecules with BWA-mem default parameters. After filtering out microbial/human DNA, and mtDNA/cpDNA from the corrected DNA (including unmapped sequences), 15.5 Gb (95.84%, 39x genome coverage) were left for estimating the R498 genome size using the following equation:

$$G = (N \times (L - K + 1) - B) / D.$$

Where N is the total number of reads, L is the average length of reads, K is k-mer length, B is the number of error kmers between zero and the dip before the hump to be discarded, D is the sequencing depth (hump) estimated from k-mer distribution, and G is the genome size.

Using 17-31 mers, the genome size was estimated to be 372.3-385.8 Mb, which was smaller than the assembled pseudomolecules. The discrepancy was caused by the inaccuracy of the equation in estimating the size of genomes with a large amount of high-copy repetitive sequences.

A total of 1 Mb of sequences was found potentially missing in the pseudomolecule regions that were covered by the genome maps, and 3 Mb centromere sequences were not incorporated into the pseudomolecules. Since the genome maps covered 96.6% of the pseudomolecules, the missing sequences throughout the genome except the centromere regions should be not much larger than 1 Mb. Considering the high redundancy (>15%) in PBcR HS assembly, we estimated the true missing centromere sequences probably to be <2.5 Mb. Meanwhile, we used the Illumina short reads to estimate the centromere gap size[2]. After alignment with BWA-mem, only one best aligned position was selected for each read, and the sequencing depth for each window of 1 kb was computed. Based on the ratio of the normalized average sequencing depth of centromere regions to the whole genome, we estimated the total size of the five centromere gaps to be 1.9 Mb.

Put them together, we estimated that <3.5 Mb (<1%) of sequences were missing in the pseudomolecules. In addition, we assembled the 38.7 Gb of Illumina short read data to 477 Mb contig sequences by SOAPdenovo (Supplementary Table 2), of which 466.2 Mb (97.74%) were aligned to the R498 pseudomolecules. It is notable that SOAPdenovo generated very few chimeric contigs, though with high sequence redundancy. After removing contaminated sequences from microbial and human genomes, only less than 1.0 Mb of non-redundant sequences were not aligned to the pseudomolecules.

Supplementary References

1. Chaisson, M. J. P., Wilson, R. K. & Eichler, E. E. Genetic variation and the de novo assembly of human genomes. *Nat. Rev. Genet.* **16,** 627–640 (2015).
2. Long, Q. *et al.* Massive genomic variation and strong selection in Arabidopsis thaliana lines from Sweden. *Nat. Genet.* **45,** 884–890 (2013).
